# Kinase signaling in liver disease *via* clinical-trial-on-a-PamChip: A distinctive methodology for drug mechanisms and personalized medicine

**DOI:** 10.1016/j.jbc.2026.111379

**Published:** 2026-03-18

**Authors:** Zachary A. Kipp, Evelyn A. Bates, Genesee J. Martinez, Wang-Hsin Lee, Sally N. Pauss, Terry D. Hinds

**Affiliations:** 1 Department of Pharmacology and Nutritional Sciences, Drug & Disease Discovery D3 Research Center, University of Kentucky College of Medicine, Lexington, Kentucky, USA; 2 Barnstable Brown Diabetes Center, University of Kentucky College of Medicine, Lexington, Kentucky, USA; 3 Markey Cancer Center, University of Kentucky, Lexington, Kentucky, USA

**Keywords:** drug discovery, biomarkers, organ-on-a-chip, side effects, cancer, HCC, MASLD, fibrosis, cirrhosis, obesity

## Abstract

The utilization of extensive datasets, such as those generated by DNA or RNA sequencing, has become a central focus in drug discovery and personalized medicine (PerMed). Nonetheless, these datasets are constrained by the absence of protein-functionality testing, which affects physiological responses. In this study, we employed PamGene PamStation technology to quantify protein function by kinase activity across more than 500 signaling pathways from patients with hepatocellular carcinoma (HCC). Using proteins derived from nine patients and five human HCC cell lines for drug discovery and the clinical trial-on-a-chip approach, we developed comprehensive PerMed scores from the PamStation data to differentiate individual kinase activity levels for each patient, stratified by sex. This methodology revealed significant responses to kinase inhibitor compounds in some HCC patient samples. In opposition, other patient protein responses to these compounds exhibited off-target signaling pathways, indicating that possible side effects, such as hypoglycemia, could occur if the patient were administered the drug. Such techniques hold potential for PerMed applications by identifying the most effective medication for each individual, identifying potential side effects, and reducing reliance on animal testing in biomedical research. Our findings contribute to the development of an applicable kinome atlas and highlight the potential of PamGene PamStation technology to advance precision medicine, including instrumented humanized clinical trial applications.

Protein phosphorylation by kinases is the most common post-translational modification, accounting for nearly one-third of all such modifications (1). Variations in phosphorylation are highly relevant to many diseases, including cancer, metabolic disorders, and endocrine conditions (2, 3, 4). Although several technologies exist to assess kinase pathways, they have limitations and are not tailored for comprehensive omics analyses of multiple pathways simultaneously. Similarly, RNA-Seq pathway analysis does not reveal the true drivers of mRNA transcripts. A thorough investigation of hepatocellular carcinoma (HCC) by The Cancer Genome Atlas Research Network analyzed 196 HCC samples for DNA methylation, RNA, miRNA, and proteomics, and 363 samples *via* whole-exome sequencing and DNA copy number analysis (5). This study identified 25 downstream pathways of receptor tyrosine kinases (TKs), whose mutation frequencies likely affect their function in HCC, underscoring the importance of these kinases in disease progression (5). This research has significantly advanced the understanding of gene expression in HCC but did not directly evaluate protein activity. Therefore, methods that directly measure protein function and signaling pathway changes are crucial for further understanding how these pathways are altered in disease.

Recent advances in research technologies have focused on elucidating kinase activity, proteins that are considered master regulators of biological functions and physiological processes, and also influence drug responses (6). The effects of kinases are opposed by phosphatases (7, 8), which remove phosphate groups and regulate downstream pathways, including HCC development and progression and drug resistance (9). Although the functions of phosphatase proteins are essential and warrant further investigation, no technologies are currently available to screen for broad phosphatase activity in real time. Commonly employed methodologies for assessing kinase activity encompass immunoblotting, microarray technologies, and phosphoarrays. A state-of-the-art kinome microarray technology from PamGene, called the PamStation, has emerged as a promising instrument for real-time kinase analysis. The difference between the PamStation and a traditional phosphoarray is that the PamStation requires the inclusion of ATP in the cell or tissue lysate and acquires images at each step of the reaction, thereby allowing real-time, simultaneous phosphorylation measurement of hundreds of substrates and kinase activities. The PamStation instrument utilizes PamChip microarray technology, which integrates experimentally validated peptide sequences incorporating kinase docking domains that facilitate substrate phosphorylation at phosphotyrosine kinase (PTK) or serine/threonine kinase (STK) sites (10), exceeding 340 substrates for kinase proteins (11). A more recent advancement in the PamGene PamStation technology is the nuclear hormone receptor PamChip, which measures interactions between nuclear receptor transcription factors and coregulator proteins (12), factors mediating gene expression and function (described further in Ref. (13)). A particularly promising application of the PamGene PamStation kinome technology is its potential to analyze these pathways in personalized medicine (PerMed).

We published a study that utilized the PamGene kinome technology to examine human subjects with cirrhosis, a condition characterized by end-stage fibrosis. In this study, we conducted a comparative analysis of their kinase pathways against three rodent models of liver fibrosis. Our findings indicated that these subjects and rodent models exhibit analogous disease-related kinase signaling pathways, with the discoidin domain receptor, a receptor TK activated by collagen (14), and the insulin receptor (INSR) identified as the two most active PTKs (2). We delineated specific kinase activities within the PTK and STK families and assessed their implications in differentiating disease states from healthy controls. Following this work, we further investigated INSR in human hepatic stellate cells, which, upon activation, drive liver fibrosis (15, 16). We found that insulin resistance initiates and stimulates fibrogenic signaling pathways in hepatic stellate cells (17). Taken together, our data indicated that this functional protein analysis and the associated data have significant potential to enhance predictions of treatment options and outcomes for HCC patients.

HCC represents the most prevalent form of primary liver cancer, occurring in over 80% of cases (18, 19). The overall 5-year survival rate for individuals diagnosed with HCC is less than 20%, with this percentage declining to below 3% for advanced stages of the disease (18, 20). Such a dismal prognosis is particularly concerning, especially given the global epidemic of hepatic dysfunction associated with obesity (21, 22), referred to as metabolic dysfunction–associated steatotic liver disease (MASLD) (22, 23, 24), which significantly increases the risk of developing HCC (25). The global prevalence of MASLD is estimated at approximately 1.6 billion individuals (26), underscoring the potential for a substantial increase in future HCC cases. Unfortunately, HCC tumors are typically identified only at advanced stages, which are not suitable for liver transplantation. The standard treatment approach for advanced HCC is multimodal, requiring a combination of pharmacological agents because of high levels of therapeutic resistance (27).

Consequently, the PamGene PamStation was employed in this study to quantify the activities of hundreds of proteins in male and female patients with HCC. We profiled real-time kinase activities by measuring the phosphorylation levels of 340 substrates quantified over 124 and 94 reaction cycles during the PamChip runs for STK and PTK, respectively. To identify key regulatory networks, we conducted upstream kinome bioinformatics analysis encompassing more than 500 kinase activities, as documented in our previous studies (2, 4, 17, 28, 29, 30, 31). It was hypothesized that integrating STK and PTK data for each patient would enable the assessment of the efficacy and potential off-target effects of various pharmacological agents across multiple diseases. Off-target effects were defined as proteins that may interact directly with the tested drug or compound, or indirectly *via* its inhibition of the kinase target, thereby altering downstream effectors of the associated substrate(s). Regardless, the results indicated modulation of specific pathways. To evaluate this hypothesis, drugs targeting the primary pathway identified in our patient-sample kinome analysis were incorporated into the PamStation analysis and allowed to interact with patient samples. The integration of omics data facilitated the development of an instrumented, PerMed approach—referred to as CToC—for a “Clinical Trial on a PamChip,” which can be customized per patient to improve therapeutic outcomes, expedite drug discovery, and address disease pathology.

## Results

### Human HCC tissue and initial kinase substrate sample analysis

**Figure 1:**
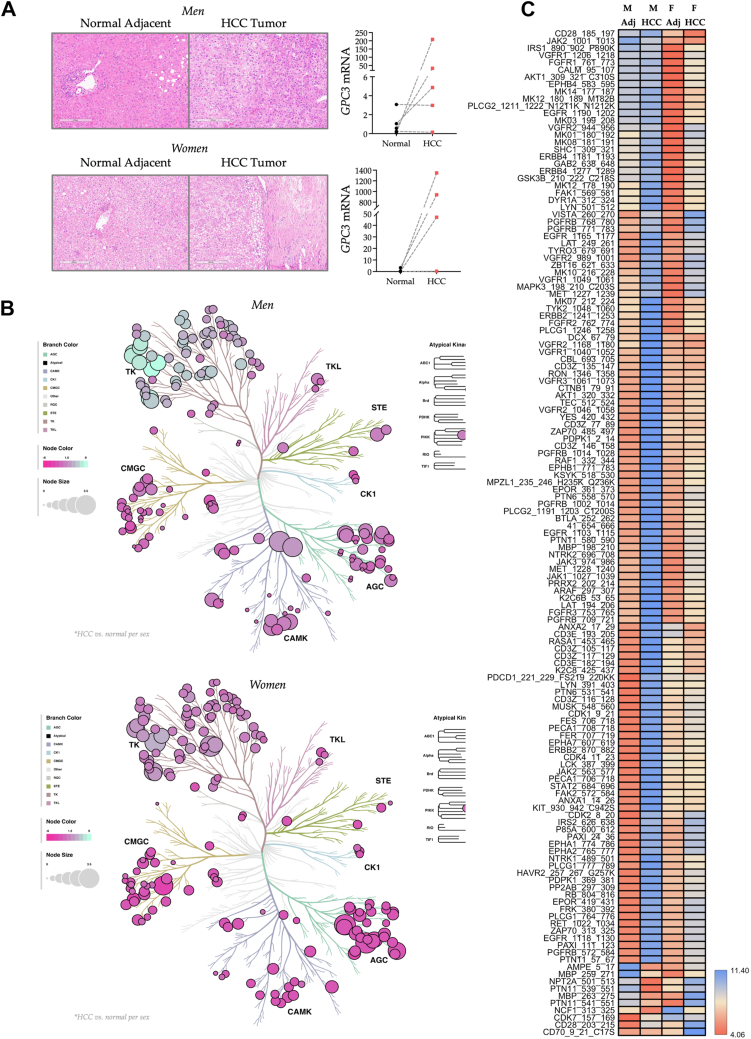
**Initial kinase activity assessment of human hepatocellular carcinoma (HCC)**. *A*, H&E images depict regions of HCC tumors from male (*top*) and female (*bottom*) subjects, including adjacent normal tissue (the scale bar represents 200 μm). In addition, *GPC3* mRNA expression was quantified in all samples (n = 9; n = 5 males, n = 4 females) as individual biological replicates. These evaluations were performed on noncancerous tissues (indicated by *black circles*) and tumor tissues (indicated by *red squares*). *B*, phylogenetic trees depict variations in PTK and STK activities in HCC tumors relative to adjacent normal tissues for males (*top*) and females (*bottom*), using pooled, equally allocated lysates analyzed in technical triplicate for group assessment, followed by individual analyses to validate the results. Node color indicates kinase activity levels, whereas node size signifies statistical significance. Branch colors denote different kinase families. *C*, a heat map presents phosphorylation levels of 196 PTK substrates in male and female HCC tumors and adjacent normal tissues across three technical replicates. PTK, phosphotyrosine kinase; STK, serine/threonine kinase.

We employed liver biopsy specimens obtained from both cancerous (HCC tumor) and noncancerous (normal adjacent tissue) regions from male and female patients diagnosed with HCC (Fig. 1*A*). The characteristics of HCC patients, including body mass index, histological data, sex, race, and plasma levels of liver dysfunction biomarkers, specifically alanine transaminase and aspartate aminotransferase, as well as the HCC biomarker alpha-fetoprotein (25), are detailed in Table S1. In addition, we validated *GPC3* expression in the specimens, a gene biomarker for HCC (32). All specimens showed increased levels in HCC tumors across both sexes, except one that had already been highly expressed in normal tissue (Fig. 1*A*).

To determine the kinase activities in these samples, we utilized the PamGene PamStation PTK and STK PamChip microarray technology. We prepared liver biopsy lysates as described for our human cirrhotic livers and for other kinome analyses (2, 4, 17, 29, 31). Here, we used female and male HCC tumors alongside normal adjacent tissue as controls. We added liver lysates to the kinome reagent mixture to identify kinase signaling differences, and we analyzed patient proteins individually or in groups in the PamGene PamStation using the PTK and STK PamChip kinome technology. We placed the calculated kinase activity data from the PTK and STK runs, totaling 340 substrates, into phylogenetic trees for visualization across sexes. Some overlaps were noted in the altered pathways for the kinase families, TKL, STE, CK1, CAMK, and CMGC (Fig. 1*B*). Combining substrate data from the PTK PamChips into a heat map reveals distinct phosphorylation levels between HCC and adjacent normal tissues in men and women (Fig. 1*C*). The adjacent tissues from both sexes exhibited similar phosphorylation levels, with distinct changes in specific substrates. Overall, the substrate phosphorylation levels in female HCC samples were lower than in male HCC samples. Nevertheless, the TK substrates were differentially phosphorylated in certain pathways in men compared with women.

### Upstream Kinase Assessments

**Figure 2:**
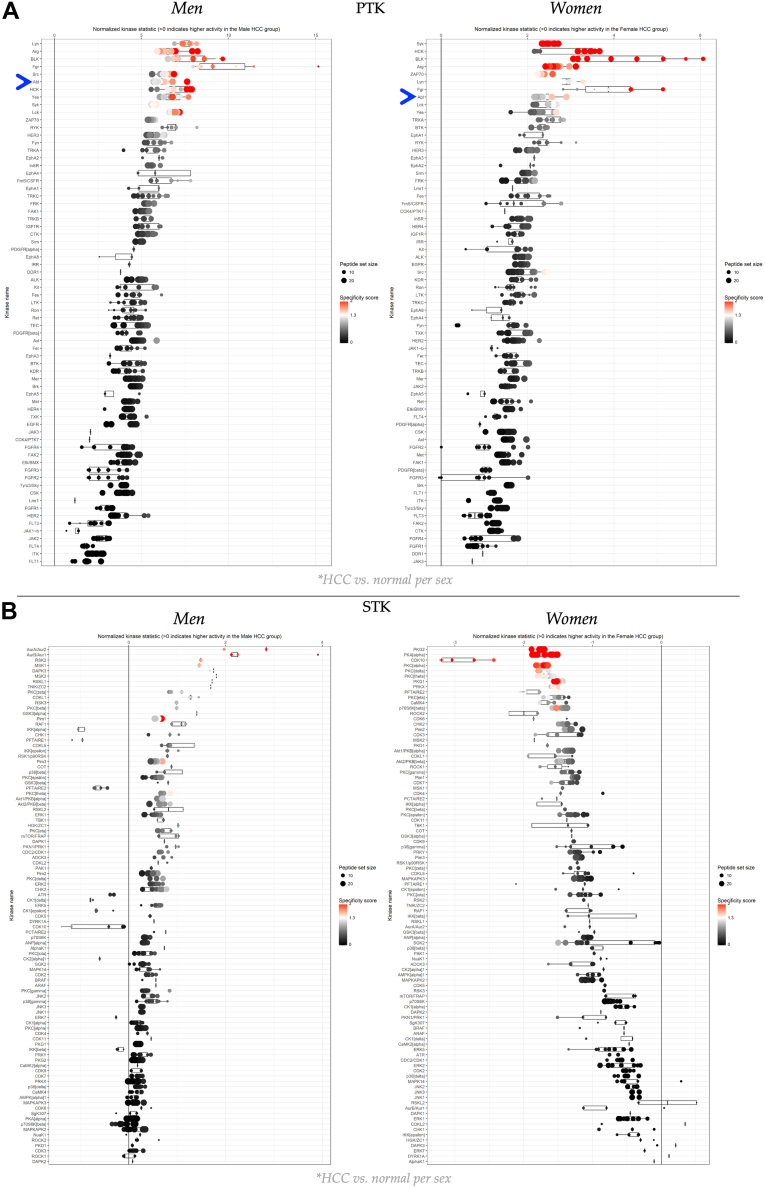
**Upstream kinase assessments in hepatocellular carcinoma (HCC) tumors**. *A*, the waterfall plot of the PTK upstream kinase analysis (UKA) illustrates kinase activity in both male and female subjects, contrasting the HCC tumor with adjacent normal tissue for each sex, based on pooled, grouped lysates. The *blue arrow* indicates the position of ABL. *B*, the waterfall plot of the STK UKA presents kinase activity in male and female subjects, comparing HCC tumor with adjacent normal tissue for each sex, utilizing pooled lysates that were analyzed in technical triplicate for group assessment, followed by individual analyses for validation. ABL, Abelson tyrosine kinase; PTK, phosphotyrosine kinase; STK, serine/threonine kinase.

We performed a more detailed bioinformatics analysis to deconvolute the kinases that promoted substrate phosphorylation using the PamGene BioNavigator Upstream Kinase Analysis (UKA) software and other methods described below. We represented the upstream kinase data derived from BioNavigator UKA in waterfall plots for the PTK and STK PamChips (Fig. 2, *A* and *B*). The BioNavigator upstream kinase data from men and women with HCC indicate that PTKs were higher in HCC than in normal adjacent controls, which are represented by the line at zero, and in comparing the top 10 kinases that exhibited the greatest changes in the waterfall plots, revealed that nine of these kinases were elevated in HCC of both sexes (Fig. 2*A*). According to the BioNavigator UKA hierarchical rating, several PTKs in the top 10 have previously been reported to play known roles in HCC. In this analysis, Abelson tyrosine kinase (ABL) activity ranked sixth among men and eighth among women (indicated by the blue arrow). ABL has a well-established role in causing HCC, and higher levels lead to worse prognosis and increased death rates (33).

The STK waterfall plot analysis revealed a dichotomy in STK signaling pathways between men and women with HCC, with men exhibiting higher STK activity and women exhibiting lower STK activity (Fig. 2*B*). Several members of the Protein Kinase C (PKC) family were elevated in males and reduced in females; some families exhibited similar regulatory actions, such as decreased PFTAIRE1 and PFTAIRE2 kinase activities in both sexes. A surprising finding was that, in men, AKT1 and AKT2 showed increased kinase activity, whereas in women they showed reduced activity. However, the PTK data for the INSR indicated higher kinase activity in both men and women (Fig. 2*A*). The changes in mRNA levels between cancerous and non-cancerous samples were mostly similar between both sexes (Fig. S1), except for GSK3, which was higher in women but not men. Validation of the expression levels of some of the most altered kinases showed that INSR, SYK, AKT1, AKT2, and ERK1 (*MAPK1*) had similar mRNA changes between normal and HCC tumor samples in both sexes, indicating that it is indeed the protein function that was considerably altered.

### Kinase hierarchical computations

**Figure 3:**
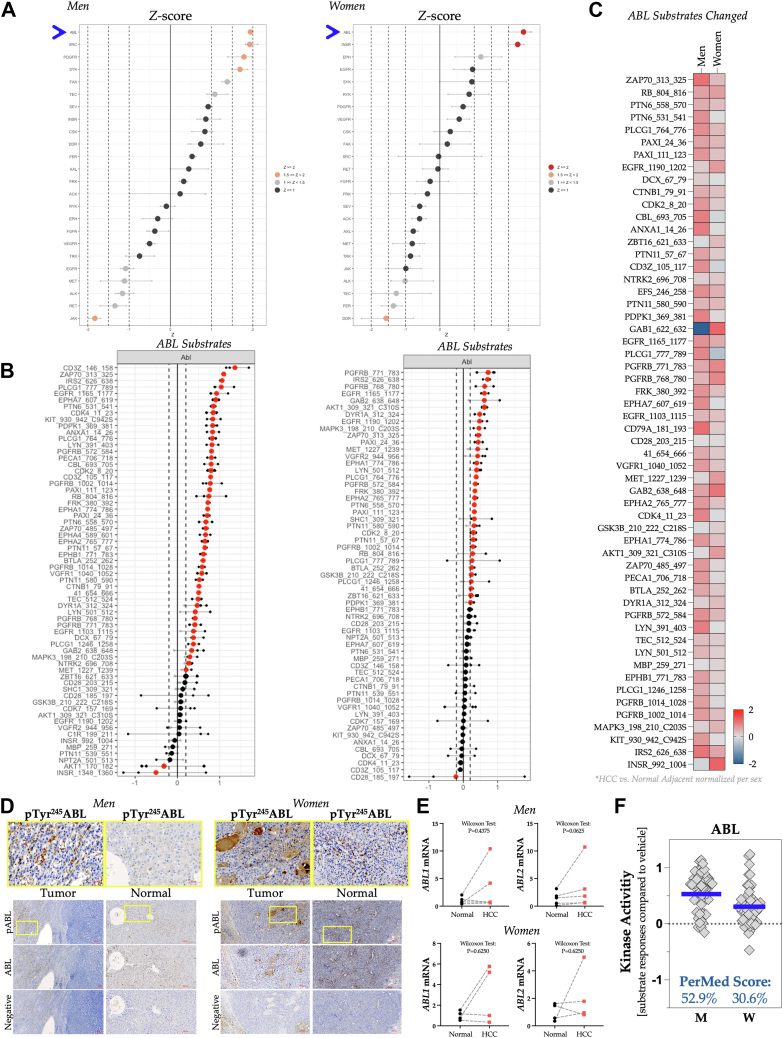
**Hierarchical kinase computations for the hepatocellular carcinoma (HCC) tumor for target pathways**. *A*, waterfall plots of Z-scores for upstream PTKs in pooled grouped HCC tumors *versus* normal adjacent tissue, displayed separately for men (*left*) and women (*right*). The *blue arrow* indicates the position of ABL. *B*, waterfall plot illustrating ABL substrates in HCC tumors compared with normal adjacent tissue in men (*left*) and women (*right*). *Red points* signify substrates passing a log fold-change threshold of 0.2. *C*, heat map depicting phosphorylation levels of ABL substrates in HCC tumors of men and women relative to normal adjacent tissue. *D*, the validation of ABL phosphorylation was performed by analyzing all samples collectively through immunohistochemistry to detect ABL tyrosine 245 phosphorylation (pTry245ABL) in HCC tumors *versus* normal tissue. The sample size was n = 9, comprising five men and four women, with each sample treated as an individual biological replicate. An ABL-specific antibody was used, along with a negative control containing only the secondary antibody, without the primary antibody. The scale bar for the *lower panels* showing pABL, ABL, and the negative control is 200 μm. A magnified view of pABL in the *upper panel* is also provided, with a 50 μm scale bar. *E*, quantitative gene analysis of *ABL1* and *ABL2* was performed with a sample size of n = 9 (men, n = 5; women, n = 4) as individual biological replicates. The results distinguish between noncancerous tissues (*black circles*) and tumor tissues (*red squares*). *F*, the kinase activity of ABL, alongside the PerMed Score, reflects the percentage change in kinase activity within the HCC tumor compared with adjacent normal tissue, based on the pooled grouped lysate analysis. The *blue line* represents the mean signal intensity for each substrate relative to the control, with the percentage change indicated above. ABL, Abelson tyrosine kinase; PerMed, personalized medicine; PTK, phosphotyrosine kinase.

To further interrogate the PTK pathways, we analyzed kinase activity data using Z-scores for each sex. The Z-score waterfall plot data analysis indicated that ABL was the most active PTK in HCC for both sexes (Fig. 3*A*). The KRSA upstream analysis also indicated ABL families as the most active kinases in men and, in women, the second-most active, after INSR (Table S2). Using the Z-score waterfall plot, INSR was the second-most active kinase in women and the eighth in men (Fig. 3*A*). On the Z-score waterfall plot, SYK ranked fourth for men and fifth among women; the KRSA analysis showed SYK ranked fourth among men and third among women. Figure 3*B* depicts a waterfall plot of the ABL substrates affected in HCC for both men and women. A heatmap comparison of ABL substrate phosphorylation levels between men and women indicates similar phosphorylation for most ABL substrates, with only a few not aligning (Fig. 3*C*).

To further investigate ABL, we conducted random-sampling analyses and quantified protein and gene expression differences between the two sexes. In Figure 3*D*, the phosphorylation of tyrosine 245 of ABL (pTyr^245^ABL) is shown to be substantially higher in the tumors of men and women with HCC, while the antibody that recognizes ABL protein expression showed no differences between tumor and non-tumor tissues for men or women. Similarly, the *ABL1* and *ABL2* mRNA levels in HCC compared with normal adjacent tissue did not differ significantly in men or women, as assessed by the Wilcoxon test for paired data (Fig. 3*E*). The PerMed score is calculated by comparing the control sample to the percentage of change in the experimental group. It reflects changes in the activity of specific kinases that may occur in disease states, during drug treatment, or as part of other investigations, for biomarker identification. The PerMed score for ABL kinase activity is derived from all ABL substrates combined for each sex, normalized to their controls (zero line), with the mean as a blue bar. The ABL PerMed score in men was 52.9% and 30.6% for women (Fig. 3*F*), indicating that ABL kinase activity in both sexes was substantially elevated in HCC.

### Drug efficacy measures *via* kinases

**Figure 4:**
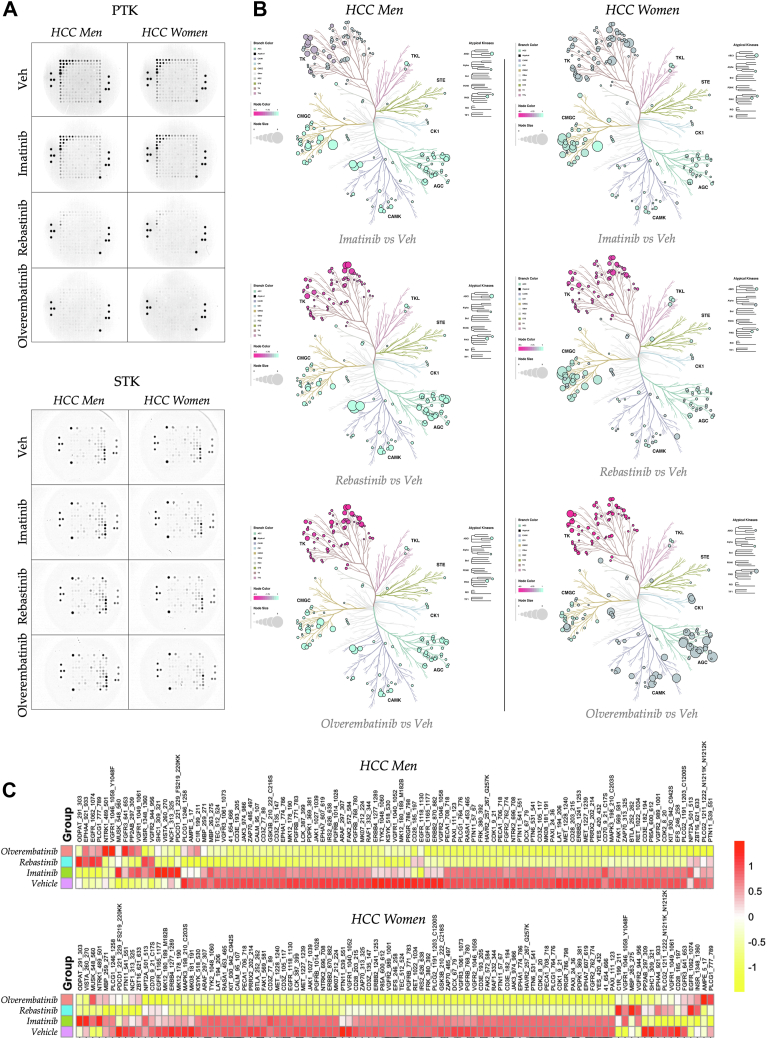
**Quantification of drug action *via* kinase and ABL inhibitor signaling**. *A*, images show phosphorylated PTK (*top*) and STK (*bottom*) PamChips during the final cycle of pooled male and female HCC tumors treated with ABL inhibitors. *B*, phyla trees depict changes in PTK and STK activity in HCC tumors treated with imatinib (*top*), rebastinib (*middle*), or olverembatinib (*bottom*), compared with a vehicle control, based on pooled tumor lysates from males (*left*) and females (*right*). Node color signifies kinase activity, whereas node size indicates significance. The branch color corresponds to the kinase family. *C*, heat maps illustrate phosphorylation levels of 196 PTK substrates across pooled HCC tumor samples from males (*top*) and females (*bottom*), each treated with vehicle, imatinib, rebastinib, or olverembatinib. ABL, Abelson tyrosine kinase; HCC, hepatocellular carcinoma; PTK, phosphotyrosine kinase; STK, serine/threonine kinase.

Based upon the finding that ABL1 was the most active kinase in HCC for both men and women, we prepared human protein lysates, promptly snap-frozen after initial sample preparation and stored at −80 °C, which were used for individual kinome analyses focusing on kinase activity in both sexes. The PTK and STK PamChip analyses were re-run, with modifications to the reagent mixture to include three ABL inhibitors: imatinib, rebastinib, or olverembatinib. The pooled HCC tumor protein lysates from both sexes were aggregated to generate a kinome atlas of drug responses for the groups (Fig. 4) or processed individually for each patient sample (Figs. S2 and S3). Hence, we used ABL inhibitors to conduct a CToC approach. A similar PamGene PamStation PTK and STK kinome running protocol was employed with two modifications. The same amount of protein lysate was used, but the ATP concentration was reduced to prevent it from outcompeting the inhibitors. The initial protein lysates were added with the same mass as before to a reagent mixture containing imatinib (1 μM final concentration), rebastinib (100 nM final concentration), olverembatinib (100 nM final concentration), or vehicle (dimethyl sulfoxide) and incubated for 30 min at room temperature while gently rocking before adding ATP and a fluorescently labeled antibody. The final concentrations of the ABL antagonist compounds were determined by their IC_50_ values described on SelleckChem.com (see the Experimental procedures section). A consistent final concentration of dimethyl sulfoxide (2%) was applied across all treatments. The operational conditions for the PamGene PamStation remained aligned with those utilized in the initial run. The resultant raw images were exported for quantification and subsequent analysis.

**Figure 5:**
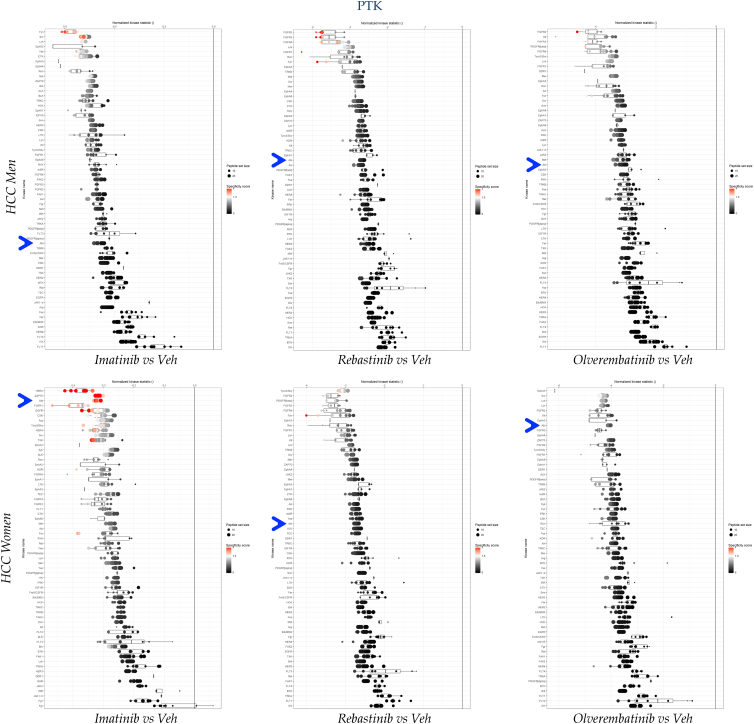
**Drug actions on kinase specificity**. A waterfall plot showing PTK upstream kinase analysis (UKA), illustrating kinase activity in both male (*top*) and female (*bottom*) subjects. The plot compares imatinib, rebastinib, and olverembatinib with vehicle control for each sex in hepatocellular carcinoma (HCC), using pooled tumor samples. The *blue arrow* indicates ABL kinase. ABL, Abelson tyrosine kinase; PTK, phosphotyrosine kinase.

The microarray phosphorylation pattern observed in the PTK PamChip exhibited a slight decrease with imatinib and a substantial reduction when treated with rebastinib or olverembatinib across both sexes (Fig. 4*A*). The phosphorylation of the STK PamChip displayed only marginal changes upon treatment with all three compounds. The integration of kinase activity data from the PamChips into phylogenetic trees for visualization of 340 substrates indicates that imatinib exerted modest effects on the TK family and had a similar influence on the STKs as rebastinib and olverembatinib (Fig. 4*B*). In conjunction with the ABL inhibitors, a heat map of PTK substrate data showed markedly reduced phosphorylation levels with rebastinib or olverembatinib. At the same time, a modest decrease in phosphorylation levels with imatinib was observed across both sexes (Fig. 4*C*). We assessed kinase activity data and represented it in waterfall plots for the PTK datasets specified in the comprehensive kinome analysis. Figure 5 depicts the specificity of the ABL inhibitors (ABL is denoted by a *blue arrow*) using the hierarchical rating system from the BioNavigator UKA software.

The activity of ABL and several PTKs were diminished; however, the STKs exhibited a dichotomous response (data not shown). The activity of multiple STKs was elevated in the presence of all three ABL inhibitors in men. The results varied in women, however, with imatinib enhancing the activity of certain STKs while diminishing that of others. Rebastinib also increased the activity of many STKs to a lesser extent than imatinib did. Conversely, olverembatinib predominantly reduced STK activity, with fewer instances of STK elevation. These findings suggest that the specificity of each compound varies, as detected using the employed methodology. Notably, pronounced sex differences were observed with all ABL antagonists. These differences will likely contribute to variation in patient outcomes, possibly because of off-target effects.

### Specific drug actions on kinases

**Figure 6:**
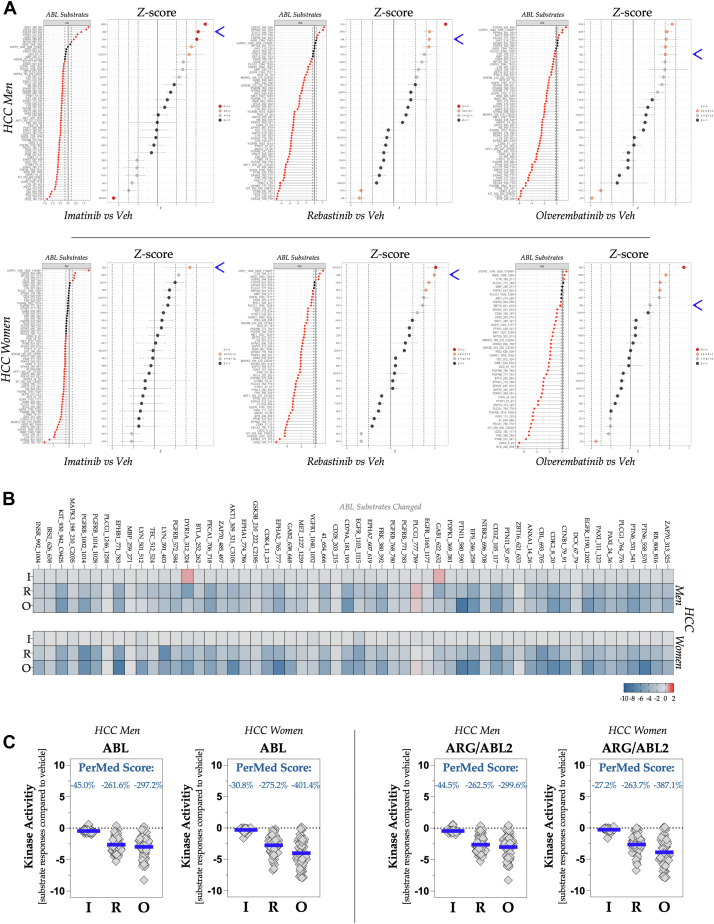
**Drug efficacy of ABL antagonists and personalized medicine (PerMed) core analysis**. *A*, the waterfall plot illustrates ABL substrates, whereas the Z-score plots compare male (*top*) and female (*bottom*) pooled, equally allocated lysates from the HCC tumor specimens. Treatments include imatinib (*left*), rebastinib (*middle*), and olverembatinib (*right*), each compared with a vehicle control. The *blue arrow* indicates the location of ABL. *Red points* in the ABL substrate waterfall plots denote substrates with a log fold change (logFC) exceeding 0.2. *B*, heat maps display phosphorylation levels of ABL substrates in males (*top*) and females (*bottom*) subjected to treatments with imatinib, rebastinib, or olverembatinib, relative to the vehicle. *C*, the kinase activity of ABL and ARG/ABL2 is assessed herein. The PerMed score represents the percentage change in kinase activity in HCC tumors from male and female subjects treated with ABL inhibitors, compared with a control group. The *blue line* denotes the mean signal intensity for each substrate relative to the control, with the percentage change annotated above. ABL, Abelson tyrosine kinase; HCC, hepatocellular carcinoma.

The waterfall plots of the ABL targets show substrates affected by the three ABL antagonist treatments, and the Z-score waterfall plot signifies that ABL families were one of the most significantly targeted kinases in the HCC kinase populations for both sexes (Fig. 6*A*). A heat map comparison of ABL substrates phosphorylated in men and women shows comparable phosphorylation across nearly all substrates, with imatinib showing weaker activity (Fig. 6*B*). Figure 6*C* shows that ABL targets changed substantially with ABL inhibitors in both men and women. Imatinib had the least effect on inhibiting ABL's activity in male and female HCC samples (PerMed score: −45.0% for men and −30.8% for women) compared with the vehicle. However, rebastinib and olverembatinib significantly reduced ABL kinase activity, with PerMed scores of −261.6% and −297.2% for men and −275.2% and −401.4% for women. An interesting observation is that imatinib had a measurably weaker inhibitory effect on proteins from HCC samples from women. Sex differences in imatinib responsiveness have been reported among patients with leukemia (34), suggesting that the molecular differences identified in our study may be related. Women have been shown to have significantly more relapse events in response to imatinib therapy (35). Our analysis may serve as an early detection tool for identifying these changes, particularly when numerous other pathway changes are used as hallmarks of resistance.

### PerMed score analysis

**Figure 7:**
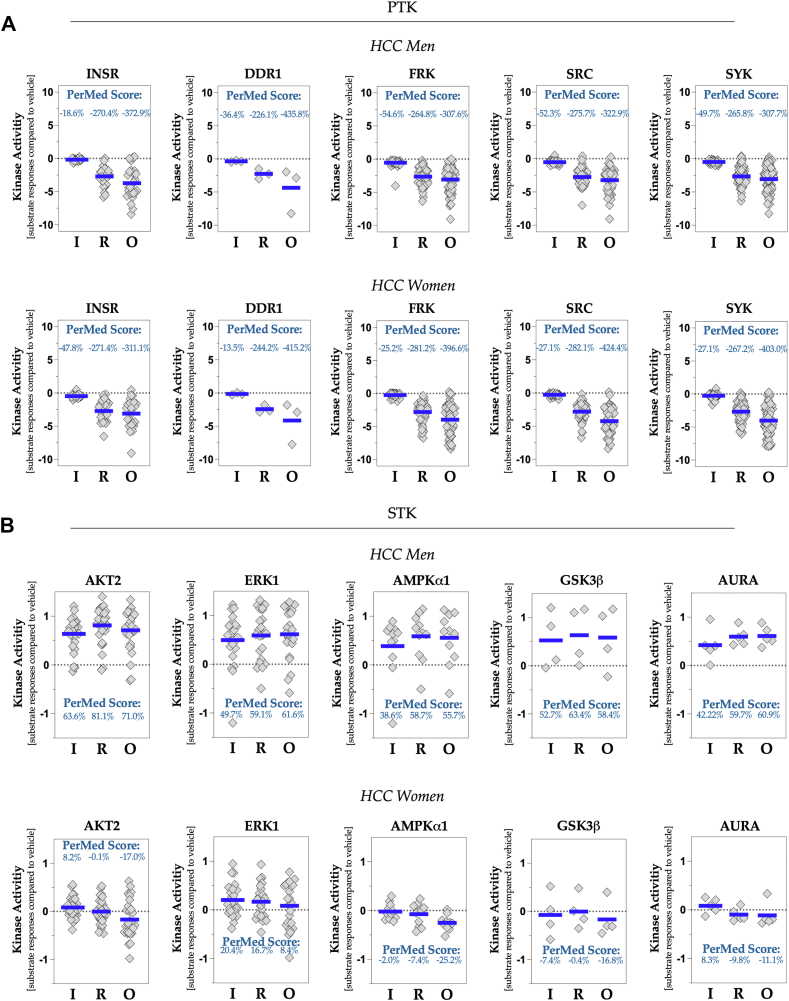
**Analysis of drug specificity and off-target effects of ABL inhibitors utilizing personalized medicine (PerMed) score methodology**. *A*, the kinase activities of PTKs, INSR, DDR1, FRK, SRC, and SYK, have been evaluated. The PerMed score quantifies the percentage change in kinase activity within pooled male and female HCC tumors following treatment with ABL inhibitors, relative to the vehicle control. The *blue line* denotes the mean signal intensity for each substrate relative to the control. *B*, in addition, the kinase activities of serine/threonine kinase (STK) family members—AKT2, ERK1, AMPK1, GSK3, and AURA—are examined. The PerMed score indicates the percentage alteration in kinase activity post-treatment with ABL inhibitors compared with the vehicle. The *blue line* illustrates the average signal intensity for each substrate in relation to the vehicle control. ABL, Abelson tyrosine kinase; DDR1, discoidin domain receptor 1; HCC, hepatocellular carcinoma; INSR, insulin receptor; PTK, phosphotyrosine kinase.

To determine how other kinase activities are affected by the three ABL antagonist treatments, we also quantified PerMed scores for other PTKs and STKs. Figure 7, *A* and *B* shows that adding ABL antagonists to the PamChip runs also altered other pathways, with some decreasing and others increasing. Inhibition of ABL kinase activity in these samples significantly increased ERK1 and AKT2 kinase activity, indicating that these pathways remain active and that changes are detectable within the kinome by adding ABL inhibitors during PamStation PamChip kinome runs. The strength of this analysis and the PerMed score lies in their ability to predict whole-kinome outcomes that lead to side effects in certain patient populations, and the sensitivity of the compound is measurable. Changes in some pathways were down in women's HCC samples but up in men's HCC samples, suggesting hallmarks of drug responsiveness that may lead to sensitivity or resistance. Using this technology to simultaneously calculate kinase activities across tens to hundreds of pathways offers a significant advantage in profiling potential drug side effects and patient outcomes.

**Figure 8:**
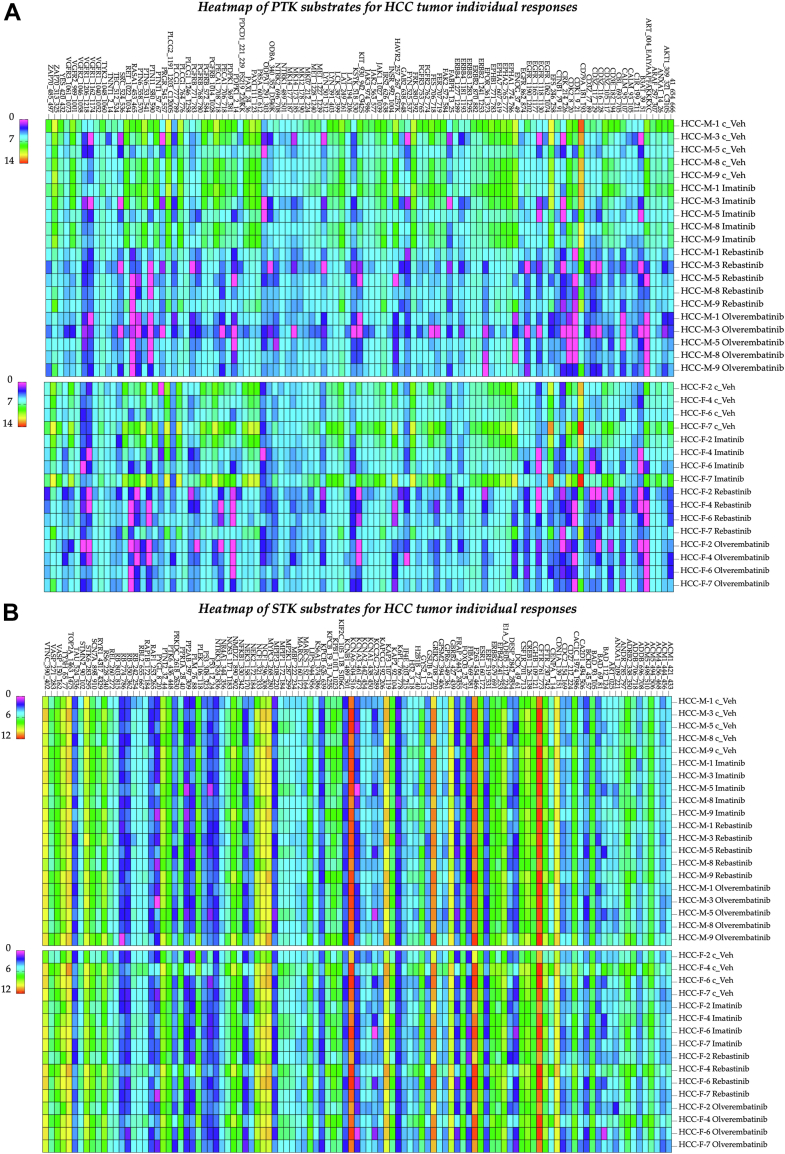
**Responses of individual HCC patients to ABL antagonists**. *A*, heat maps illustrating the PTK substrates correlated with patient-specific HCC tumor responses to both vehicle controls and ABL inhibitors in male patients (*top*) and female patients (*bottom*). (n = 9, conducted as biological replicates and individually validated). *B*, heat maps displaying the STK substrates associated with patient-specific HCC tumor responses to both vehicle controls and ABL inhibitors in male patients (*top*) and female patients (*bottom*). (n = 9, conducted as biological replicates and individually validated). ABL, Abelson tyrosine kinase; HCC, hepatocellular carcinoma; PTK, phosphotyrosine kinase; STK, serine/threonine kinase.

These methods are useful for group analysis to identify overall responses to a compound in drug discovery, or for individual analysis in PerMed. Heat map analysis of individual reactions to PTK and STK substrate phosphorylation indicates the efficacy of the compounds per patient sample (Fig. 8).

### Validation of drug actions on kinases

**Figure 9:**
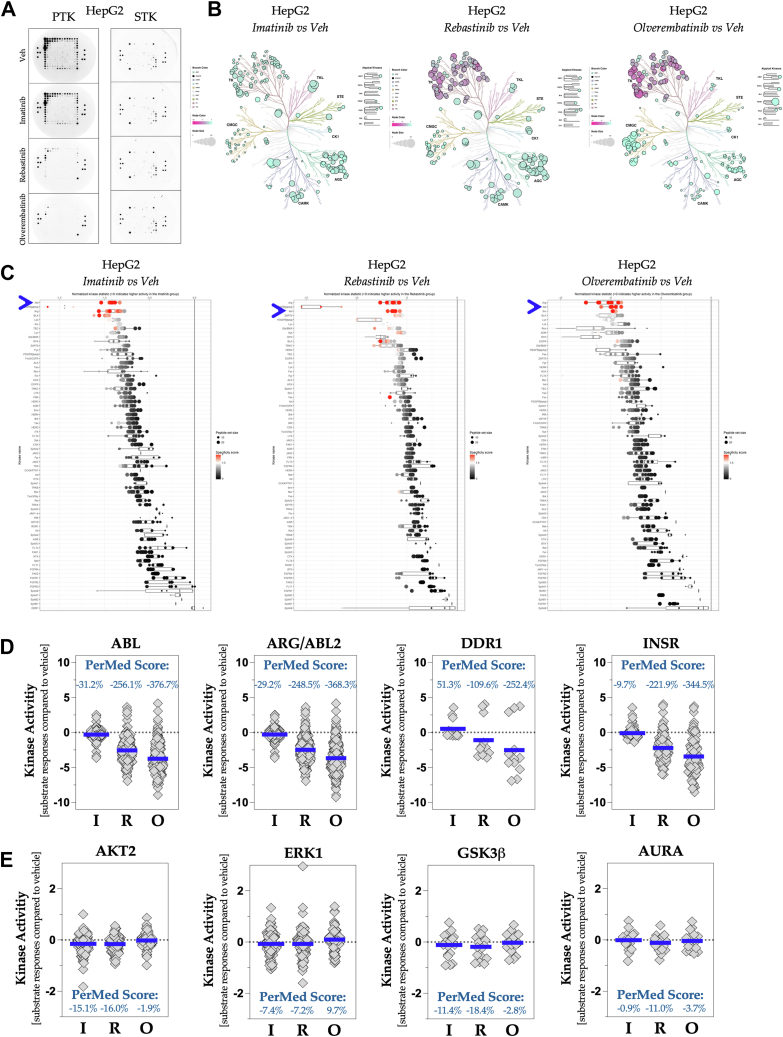
**Validation of ABL antagonist effects on kinases**. *A*, images of phosphorylated PTK (*left*) and STK (*right*) PamChip during the final cycle utilizing HepG2 cells treated with vehicle, imatinib, rebastinib, or olverembatinib (n = 3, ran as biological replicates and validated individually). *B*, phylogenetic trees illustrating the modifications in PTK and STK activity in HepG2 cells following treatment with imatinib (*left*), rebastinib (*middle*), or olverembatinib (*right*), compared with the control. Node coloration indicates levels of kinase activity, whereas node size reflects statistical significance. Branch coloration signifies kinase family associations. *C*, waterfall plot of the PTK upstream kinase analysis (UKA), depicting kinase activity in HepG2 cells treated with imatinib (*left*), rebastinib (*middle*), or olverembatinib (*right*), in comparison to the vehicle. The *blue arrow* indicates the position of ABL. *D*, the kinase activity levels of ABL, ARG/ABL2, DDR1, and INSR are presented herein. The personalized medicine (PerMed) score represents the percentage change in kinase activity in HepG2 cells subjected to ABL inhibitors relative to the vehicle control. The *blue line* shows the average signal intensity of each substrate relative to the control. *E*, the kinase activity levels of AKT2, ERK1, GSK3, and AURA are also shown. The PerMed score indicates the percentage change in kinase activity in HepG2 cells treated with ABL inhibitors compared with the control. The *blue line* denotes the average signal intensity of each substrate against the control. ABL, Abelson tyrosine kinase; DDR1, discoidin domain receptor 1; INSR, insulin receptor; PTK, phosphotyrosine kinase; STK, serine/threonine kinase.

To validate our findings in HCC samples, we employed the human HCC HepG2 hepatocyte cell line, originally isolated from a liver biopsy of a 15-year-old Caucasian male diagnosed with a well-differentiated hepatoblastoma (36). We extracted proteins from HepG2 cells and treated them with the same three ABL inhibitors to determine whether we could replicate our findings in human HCC samples. The PTK PamChip phosphorylation exhibited an almost identical pattern of responsiveness to the ABL antagonist when comparing the HepG2 cell proteins with HCC samples from both sexes (Fig. 9*A*). The visualization of HepG2 protein kinase activity responses to ABL inhibitors, as shown in phylogenetic trees, also resembled the responsiveness observed in HCC samples from men and women (Fig. 9*B*). The BioNavigator upstream analysis identified ABL and ARG/ABL2 as the kinases targeted by the compounds, indicating inhibition of PTKs relative to vehicle controls (Fig. 9*C*). The PerMed score analysis for the PTKs showed −31.2% suppression of ABL and −29.2% of ARG/ABL2 when treated with imatinib (Fig. 9*D*), which was more aligned with the responses of women's HCC protein samples. In addition, discoidin domain receptor kinase activity increased in response to imatinib; however, it declined with rebastinib and olverembatinib. The STKs displayed only modest variations in AKT2, ERK1, GSK3, and AURA activities (Fig. 9*D*). Several STKs were suppressed following treatment with imatinib and rebastinib, whereas olverembatinib produced a divergent STK response, resulting in some kinases exhibiting increased activity, whereas others demonstrated a decrease (Fig. S4).

By employing an additional four human HCC cell lines, we have corroborated these findings using HCC samples from male and female patients, in conjunction with analysis of HepG2 cell proteins. We observed that cells from male and female subjects exhibited responses analogous to those observed in the kinase activities of male and female HCC patient samples (Figs. S5 and S6). The ABL antagonists were tested using protein extracted from four additional human hepatocyte cell lines, including the human Hep3B2 HCC cell line derived from an 8-year-old Black male, established concurrently with the human HepG2 HCC cells (36) (Fig. S5). Furthermore, we evaluated protein extracts from human HLE HCC cells sourced from a 68-year-old male patient from Japan (37), Huh-7, which was ascertained from a 57-year-old Asian male patient (38), and HepaRG cells derived from a tumor of a female patient from France, with HCC resulting from a hepatitis C infection (39) (Figs. S5 and S6). These results validate our findings from the HCC tumor protein lysates, which were processed individually for each patient sample and sex (Figs. S2 and S3).

**Figure 10:**
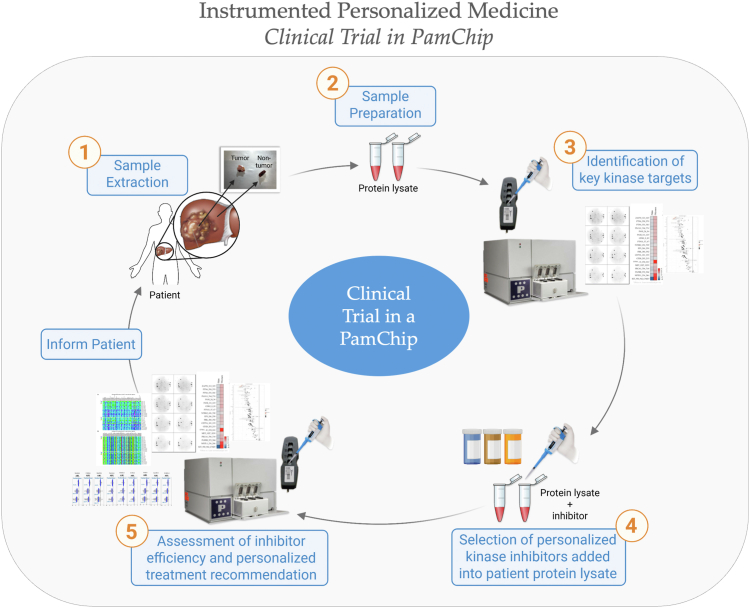
**Instrumented personalized medicine procedure**. Tissues are collected during a biopsy, and protein lysates are prepared to identify kinases. Initial profiling with the PamGene PamStation detects overactive or underactive pathways, whereas bioinformatics analysis identifies key kinase targets for each patient. Based on these targets, Food and Drug Administration–approved kinase inhibitors (or agonists) are added to the reagent mixture to assess the patient’s response. Kinase activity is reprofiled in the presence of inhibitors or agonists to evaluate effectiveness. These data assist in determining the most appropriate treatment likely to produce the optimal response.

Essentially, ABL substrate specificity revealed the depth of pathways detectable with patient-specific proteins, which can be used to discern drug efficacy or disease pathological features. These technological advancements can be incorporated into software for an instrumented, humanized “Clinical Trial in a PamChip” or personal medicine (Fig. 10), informing the patient about the best treatment options.

∗Part of this figure was created with BioRender.com.

## Discussion

In this work, we have made significant contributions to advancing kinome technology, which the PamStation holds considerable promise for the future of innovative PerMed and instrumented humanized clinical trials. This technology's primary advantage is its ability to obtain signaling data from a patient’s biopsy or cells, thereby elucidating alterations in kinase activity. As demonstrated in our findings, this information could be employed to select the optimal medication for the individual. This process can assess multiple kinase inhibitors using the same lysate from the biopsied sample to identify the most effective drug for the patient. Implementing this approach can significantly reduce the time required to establish the appropriate treatment by precisely targeting kinase pathways and identifying the most effective antagonist or agonist. Adopting PamGene technology in PerMed benefits nearly all patients and the health care system by enabling the measurement of hundreds of kinase activities for predictive testing, biomarker development, drug response monitoring, therapeutic development, and PerMed. The future of the PamGene PamStation technology is in its ability to predict signaling mechanisms in diseases and disorders and, most importantly, responses to treatments.

The observation that ABL was identified as the most active PTK through the Z-score waterfall plot is not unprecedented, as patients with HCC typically exhibit elevated ABL activity (33). Proteomics analysis by the Clinical Proteomics Tumor Analysis Consortium of 165 patients showed increased ABL protein expression in HCC tumor tissue compared with normal tissue (40, 41). This is supported by The Cancer Genome Atlas data showing a significant increase in *ABL1* mRNA in HCC tumors (n = 371) compared with normal tissue (n = 50) (40, 41). Multiple HCC cohort studies have found that high *ABL1* mRNA expression in tumors, but not in adjacent normal tissue, is associated with poorer survival (42, 43). Several studies have reported increased ABL expression; however, our analysis did not detect a significant increase in expression but rather a modification in its phosphorylation and kinase activity relative to target substrate proteins.

Importantly, pathways that ABL inhibits, such as ERK1, AKT2, and AMPKα1, demonstrated increased kinase activity in the presence of certain ABL antagonists. ABL has been shown to signal through kinases, including the PI3K pathway, which regulates AKT phosphorylation and insulin-induced glucose uptake (6, 24). In 2002, Klejman *et al*. (44) demonstrated that combining pharmacological PI3K antagonists with ABL inhibitors, such as imatinib, enhanced antileukemic effects in Philadelphia chromosome–positive chronic myelogenous leukemia and acute lymphocytic leukemia cells. Moose *et al*. (45) found that ABL kinases directly regulate AKT phosphorylation and that suppression of ABL kinases *via* siRNA knockdown or imatinib treatment altered pAKT detection. Furthermore, the use of AKT inhibitors demonstrated that ABL signaling *via* AKT1 contributes to tumor cell growth. ABL may also regulate energy signaling pathways, as Vakana *et al*. (46) demonstrated that AMP-activated protein kinase and ABL communicate through treatment with the AMP-activated protein kinase activator 5-aminoimidazole-4-carboxamide ribonucleotide and ABL antagonist imatinib. They also showed that 5-aminoimidazole-4-carboxamide ribonucleotide was more effective at inhibiting imatinib-resistant BV173R Philadelphia chromosome–positive acute lymphocytic leukemia cell viability than in chronic myelogenous leukemia, and that metformin had minimal effects on both. Wu *et al*. (47) demonstrated that inhibition of ABL kinase activity with ON012380 disrupted other pathways, thereby affecting the phosphorylation of STAT5, CrkL, HCK, and LYN kinases in BaF3 murine pro-B lymphocyte cells that expressed either wildtype or imatinib-resistant BCR-ABL. These could indicate off-target drug effects.

The off-target effects in our study were quantifiable across hundreds of kinase signaling pathways. ABL antagonists have been documented to alter glucose sensitivity in some patients but not others. Gómez-Sámano *et al*. (48) demonstrated that individuals diagnosed with chronic myeloid leukemia or gastrointestinal stromal tumors who also have type 2 diabetes and are treated with the ABL inhibitor imatinib experience a significant reduction in fasting plasma glucose and a decrease in glycosylated hemoglobin A1C. ERK1 and AKT2 are critical components of the insulin signaling cascade (24), and their increased signaling activity was evident with ABL inhibitors in the PamChip kinome assay, providing valuable insight into off-target drug effects. Consequently, these data support using the “Clinical Trial in a PamChip” methodology to ascertain the efficacy of agonists and antagonists from a specific patient before treatment to detect off-target signaling accompanying side effects, such as hypoglycemia. In this study, the effects of the inhibitors on the PTK family are likely because of direct inhibition of ATP binding, as they all bind to conserved sites within the catalytic domain, thereby strongly inhibiting PTK substrate phosphorylation (45, 49). There was no strong inhibition of STK substrate phosphorylation with the inhibitors used, suggesting that the observed effects may be mediated by indirect mechanisms. The effectiveness of each compound can be quantified, and outcomes within the respective pathways can be assessed prior to patient administration.

PerMed is critical for preventing, diagnosing, and treating diseases with diverse pathologies within affected populations. Omics techniques utilized in PerMed, such as DNA sequencing and its derivatives (RNA-Seq, chromatin immunoprecipitation sequencing, and assay for transposase-accessible chromatin using sequencing, among others), have proven essential for comprehending the pathology of numerous diseases and metabolic states (metabolomics), which include various forms of cancer, MASLD, obesity, and cardiovascular diseases, and apply to nearly all disorders and diseases. Notwithstanding advancements in RNA-Seq and related technologies that report transcript abundance, these methods are limited in their ability to provide insights into disease- or drug-related functional pathways. Protein translation and activity are moderated by transcription factor activity (13), post-translational modifications, and other regulatory mechanisms that influence protein stability, and mRNA expression may differ from protein levels (50). For these reasons, establishing causal relationships solely on the basis of transcript abundance data derived from RNA-Seq is challenging and impractical for determining drug selectivity in patient samples. Our multiomic kinome technique (Fig. 10), designed to elucidate the complex mechanisms of disease and drug action, holds significant promise for research applications and could revolutionize clinical practice.

Omics data analysis underpins real-time kinase activity profiling with the PamGene. Given the nature of the measurement, a direct identification of which kinase contributes to the phosphorylation pattern of the 340 substrates is not readily accessible. Rather, it is derived through a pathway deconvolution process that generally aggregates all known peptide motifs that a kinase can phosphorylate. Subsequently, the data are compared with the phosphorylation pattern on the PamChip to identify the kinases most likely responsible for those phosphorylation events. The design of the PamGene PamChip kinome assays facilitates comprehensive omics data analysis, enabling users to assess protein function in disease and drug therapies, particularly when multiple signaling pathways are disrupted.

The PamGene kinase activity profiling represents an innovative instrument that has already demonstrated utility in oncological research (51, 52, 53), liver fibrosis and cirrhosis (2, 17, 29), and metabolic diseases (2, 4, 28, 30, 31, 54). Oxidative stress induced by reactive oxygen species in hepatic cells initiates lipid accumulation (55, 56, 57), which may lead to MASLD (6, 23) and, if persistent, progress to metabolic dysfunction–associated steatohepatitis (2, 15, 17, 29), wherein liver fat accumulation (steatosis) results in sustained inflammation and hepatocyte injury (23). This constitutes a more severe stage characterized by potential fibrosis (scarring), thereby increasing the risk of cirrhosis, hepatic failure, and HCC (23). An intriguing aspect of liver dysfunction concerns plasma bilirubin levels, which are predominantly, though not exclusively, reduced in individuals with metabolic diseases (58) yet elevated in nearly all patients suffering from cirrhosis (2) or HCC (59). The phenomenon of inverse bilirubin levels is likely attributable to the expression of uridine diphosphate glucuronosyltransferase 1A, an enzyme responsible for bilirubin conjugation, thus facilitating its removal from the bloodstream (4, 21, 57, 60). Notably, Shinn *et al*. (61) observed that bilirubin inhibits liver fibrosis and demonstrated that bilirubin-based nanoparticles may serve as potential therapeutics for the disease.

In a prior investigation, we performed bioinformatic analyses of pathways in liver fibrosis, where we deconvoluted kinase signaling pathways using PamGene kinome and RNA-Seq technologies (17, 29). In separate publications using both technologies, we demonstrated that FOXS1 and INSR modulate fibrosis by regulating hepatic fibrotic signaling (17, 29). We used RNA-Seq and the PamGene kinome analysis to elucidate the pathways they govern, and we observed similar findings across the two datasets. Others have profiled the kinome of peripheral blood mononuclear cells derived from patients with melanoma or non–small cell lung cancer treated with two distinct immune checkpoint neutralizers (53). The researchers successfully developed a predictive model using kinome data to classify treatment groups with up to 100% accuracy in the melanoma patient population (53). In addition, other studies have assessed normal and malignant tissues from patients with renal cell carcinoma and their responses to four PTK inhibitors. They identified 36 kinases whose activity differed significantly between normal and cancerous tissues. Following *ex vivo* treatment, tivozanib and cabozantinib showed a more pronounced inhibitory effect on kinase pathways in cancerous tissues than sunitinib and pazopanib (51). Our study was not *ex vivo*, as the drugs were added directly to the reagent mixtures and then adjusted for PamChip operation. Our study developed a method that others can use to better understand disease mechanisms, without the need to treat a living person to determine responsiveness, with the hope of improving treatment regimens and outcomes.

In this study, numerous members of the TK family showed hyperkinase activity in HCC tumors in both sexes, as determined by PamGene technology. Our *Z*-score analysis in this study showed that ABL had the highest kinase activity in HCC tumors in both sexes, despite unchanged mRNA levels. Most interesting and promising for PerMed, ABL antagonism in HCC specimen lysates not only reversed intended signaling pathways but also identified changes in other pathways that can serve as hallmarks of off-target signaling leading to drug side effects. In rare cases, it can identify a patient who does not respond to the drug as expected. This is shown in Figure S2*B*, where patient HCC-F-7 displayed increased ABL activity with imatinib. This surprising data point could significantly affect the patient's treatment and outcome. If treated with imatinib, the patient’s condition may worsen. Imatinib has been shown to act as an allosteric activator of ABL by binding to a secondary myristoyl pocket, albeit with lower affinity than the catalytic site (49). The binding of imatinib to this secondary site may be favored in patients with mutations in the catalytic domain (49). The power of this technology lies in its ability to determine protein function in the presence of inhibitors, without the need for genetic sequencing to identify mutations that may or may not affect function. Proteins are the ones affected by SNPs, and this technology enables testing of their functions. However, gene sequencing analysis has been shown to be beneficial for some treatments (62, 63). The PamGene technology enables the prediction of a patient’s treatment response and, therefore, facilitates treatment planning, improving the patient’s chances of success.

Here, we present a kinomic atlas for human HCC tumors, validating our findings in human HCC samples and cells. This endeavor may significantly enhance the scientific understanding of the mechanisms underlying HCC. This work extends the capabilities of the PamGene PamStation technology to incorporate drug compounds, such as ABL antagonists, and the method can also be used to test other compounds across different diseases and tissues. When we combined the HCC specimens with the ABL inhibitors and re-ran the same lysate samples, we detected significantly lower ABL kinase activity in the presence of the antagonists. Even though there was no intact cell signaling system, the samples consisted of lysates with residual kinase activity remaining. The bioinformatics analyses demonstrated the effectiveness and sensitivity of these compounds, revealing alterations in numerous pathways despite the absence of intact cells. Observing an overactive ABL kinase in HCC was not novel; however, the concurrent use of ABL antagonists while examining and running the PamChip kinome in these samples has not been reported previously.

In conclusion, the PamStation kinome technology demonstrates significant clinical utility; however, its implementation in clinical environments is limited by factors, such as cost, usability, and data analysis challenges. The PamGene PamStation technology offers numerous practical applications through the CToC approach, which helps elucidate disease pathways, drug mechanisms, and PerMed. This methodology, with a focus on instrumented PerMed, improves the capacity to classify patient subtypes based on kinase signaling pathways, thereby potentially enhancing treatment responsiveness and sensitivity. Our advancements in PamGene kinase technology may serve as diagnostic tools for identifying the causes and mechanisms underlying diseases. The use of the PerMed Score, coupled with artificial intelligence software, facilitates the identification of patterns in changes in kinase activity. This software could revolutionize the understanding of disease processes, drug actions, and adverse effects, ultimately improving clinical outcomes. An advantage of this study is the quantification of the kinome atlas through profiling kinase activities across hundreds of pathways. In addition, we have developed PerMed technology that provides insights into the efficacy of therapeutic interventions for individual patients, including side-effect profiles, thereby enhancing treatment success and quality of life. Ultimately, these advancements hold the potential to produce more effective therapeutics tailored to an individual’s protein responsiveness, thereby improving both patient experience and overall treatment success.

## Experimental procedures

### Human subjects

The institutional review board (IRB) at the University of Kentucky waived ethical approval for this work because the human liver samples were deidentified, and the authors had no access to patient identifiers. The sample names were randomly assigned to ensure the results could not be linked to a patient. The University of Kentucky IRB deemed this project did not require full IRB review because it fulfills the “United States Department of Health & Human Services 2008 Coded Private Information or Specimen Use in Research Guidance” requirements for this decision. All other methods and procedures are described in more detail in the STAR★METHODS. All studies using human tissue samples conformed to the ethical guidelines of the 1975 Declaration of Helsinki, as reflected in approval by the Human Subjects Committee of the University of Kentucky College of Medicine.

## Data availability

### Lead contact

Requests for resources and further information should be directed to the lead contact, Dr Terry Hinds (Terry.Hinds@uky.edu).

### Materials availability

This study did not generate any new unique reagents.

### Data and code availability

The kinase reports and raw files will be available on Figshare (URL 10.6084/m9.figshare.31129486) at the publication date. This study did not generate any unique code.

## Supporting information

This article contains supporting information (2, 29, 30, 31).

## Conflict of interest

Z. A. K., E. A. B., and T. D. H. have submitted utility patents on personalized medicine and humanized clinical trial applications. T. D. H. has patented the use of bilirubin nanoparticles in cardiometabolic disorders (patent number: WO2020176289A1). The authors declare that they have no conflicts of interest with the contents of this article.
