# Supplementary material for: Kinase signaling in liver disease via clinical-trial-on-a-PamChip: A distinctive methodology for drug mechanisms and personalized medicine
Source: J Biol Chem. 2026 Mar 18;302(5):111379. doi: 10.1016/j.jbc.2026.111379 (PMC13091354; doi:10.1016/j.jbc.2026.111379)
Supplement: Supplementary Material 1 [file mmc1.docx]

**SUPPLEMENTAL TABLES**

**Supplemental Table 1.** Liver Biopsy Patient Clinical Characteristics (expressed as: average (standard deviation)).

|  | Male | Female |
| --- | --- | --- |
| N # | 5(9) | 4(9) |
| Age | 65 (7.52) | 68.25 (9.22) |
| BMI | 29.22 (4.97) | 32.4 (9.18) |
| Tumor Nuclei (%) | 88% (10.37) | 83.75% (11.09) |
| Hepatitis Status (%) | 20% | 0% |
| ALT (U/L) | 434.80 (713.46) | 183.75 (210.33) |
| AST (U/L) | 264.33 (419.73) | 280.00 (428.71) |
| AFP (ng/mL) | 7.27 (8.37) | 8849.60 (17549.71) |

**Supplemental Table 2A.** Phospho-Tyrosine Kinase (PTK) - Male HCC vs Male Adjacent Normal Upstream Kinase Analysis (UKA) utilizing pooled lysates analyzed in technical triplicate for group assessment - Kinase Families.

| Kinase | Observed | SamplingAvg | SD | Z |
| --- | --- | --- | --- | --- |
| **ABL** | 43 | 36.582 | 3.203 | 2.004 |
| PDGFR | 84 | 77.930 | 3.086 | 1.967 |
| SRC | 97 | 92.185 | 2.634 | 1.828 |
| SYK | 79 | 73.218 | 3.396 | 1.703 |
| JAK | 14 | 18.267 | 2.530 | -1.686 |
| FAK | 37 | 32.522 | 3.088 | 1.450 |
| TEC | 84 | 79.702 | 3.093 | 1.389 |
| ALK | 25 | 28.969 | 2.988 | -1.328 |
| DDR | 24 | 20.661 | 2.594 | 1.287 |
| RET | 5 | 7.064 | 1.636 | -1.261 |
| INSR | 22 | 18.952 | 2.513 | 1.213 |
| EGFR | 72 | 76.162 | 3.454 | -1.205 |
| CSK | 44 | 40.698 | 3.234 | 1.021 |
| AXL | 37 | 34.190 | 3.053 | 0.921 |
| ACK | 1 | 0.586 | 0.493 | 0.841 |
| SEV | 1 | 0.588 | 0.492 | 0.837 |
| EPH | 20 | 21.850 | 2.766 | -0.669 |
| VEGFR | 17 | 18.375 | 2.516 | -0.546 |
| FER | 6 | 5.320 | 1.450 | 0.469 |
| MET | 19 | 20.160 | 2.599 | -0.446 |

**Supplemental Table 2B.** Phospho-Tyrosine Kinase (PTK) - Female HCC vs. Female Adjacent Normal Upstream Kinase Analysis (UKA) using pooled lysates, analyzed in technical triplicate for group assessment - Kinase Families.

| Kinase | Observed | SamplingAvg | SD | Z |
| --- | --- | --- | --- | --- |
| INSR | 19 | 12.743 | 2.549 | 2.455 |
| **ABL** | 31 | 24.720 | 3.128 | 2.008 |
| SYK | 56 | 49.556 | 3.325 | 1.938 |
| SRC | 59 | 62.264 | 2.705 | -1.206 |
| FER | 2 | 3.595 | 1.449 | -1.100 |
| FRK | 7 | 5.150 | 1.719 | 1.076 |
| AXL | 20 | 23.146 | 3.052 | -1.031 |
| TRK | 6 | 7.987 | 2.073 | -0.959 |
| ACK | 0 | 0.401 | 0.490 | -0.818 |
| SEV | 0 | 0.390 | 0.488 | -0.800 |
| DDR | 12 | 14.050 | 2.670 | -0.768 |
| PDGFR | 55 | 52.594 | 3.161 | 0.761 |
| EGFR | 54 | 51.612 | 3.361 | 0.711 |
| VEGFR | 14 | 12.456 | 2.541 | 0.608 |
| FAK | 20 | 21.837 | 3.110 | -0.591 |
| MET | 12 | 13.518 | 2.577 | -0.589 |
| JAK | 11 | 12.363 | 2.518 | -0.541 |
| RET | 4 | 4.826 | 1.658 | -0.498 |
| EPH | 16 | 14.780 | 2.774 | 0.440 |
| TEC | 53 | 53.846 | 3.194 | -0.265 |
| FGFR | 21 | 20.270 | 2.944 | 0.248 |
| RYK | 1 | 0.831 | 0.695 | 0.243 |
| ALK | 19 | 19.549 | 2.955 | -0.186 |
| CSK | 27 | 27.568 | 3.312 | -0.171 |

**Supplemental Table 3A.** Serine-Threonine Kinase (STK) - Male HCC vs Male Adjacent Normal Upstream Kinase Analysis (UKA) utilizing pooled lysates analyzed in technical triplicate for group assessment - Kinase Families.

| Kinase | Observed | SamplingAvg | SD | Z |
| --- | --- | --- | --- | --- |
| HCK | 34 | 26.059 | 2.896 | 2.742 |
| Yes | 31 | 23.542 | 2.745 | 2.717 |
| HER2 | 28 | 36.551 | 3.211 | -2.663 |
| ALK | 40 | 47.320 | 3.273 | -2.237 |
| FRK | 51 | 43.596 | 3.337 | 2.219 |
| Tyk2 | 1 | 3.558 | 1.164 | -2.198 |
| Lck | 31 | 24.721 | 2.862 | 2.194 |
| JAK1 | 1 | 3.562 | 1.173 | -2.184 |
| EphB1 | 1 | 3.538 | 1.174 | -2.162 |
| Lyn | 23 | 17.616 | 2.591 | 2.078 |
| FAK1 | 41 | 47.910 | 3.337 | -2.071 |
| BLK | 27 | 21.772 | 2.682 | 1.949 |
| HER4 | 29 | 34.742 | 3.041 | -1.888 |
| EphA7 | 1 | 2.990 | 1.076 | -1.850 |
| FGFR3 | 9 | 12.993 | 2.167 | -1.842 |
| Src | 40 | 34.205 | 3.194 | 1.814 |
| EphB2 | 1 | 2.973 | 1.114 | -1.770 |
| EphB3 | 1 | 2.934 | 1.093 | -1.770 |
| EGFR | 36 | 41.980 | 3.394 | -1.762 |
| JAK3 | 3 | 5.329 | 1.405 | -1.658 |
| Ros | 4 | 2.394 | 1.001 | 1.605 |
| KDR | 13 | 16.539 | 2.428 | -1.458 |
| FGFR2 | 11 | 14.190 | 2.257 | -1.413 |
| FLT4 | 10 | 12.985 | 2.149 | -1.389 |
| GSK3[beta] | 0 | 0.615 | 0.487 | -1.264 |

**Supplemental Table 3B.** Serine-Threonine Kinase (STK) - Female HCC vs Female Adjacent Normal Upstream Kinase Analysis (UKA) utilizing pooled lysates analyzed in technical triplicate for group assessment - Kinase Families.

| Kinase | Observed | SamplingAvg | SD | Z |
| --- | --- | --- | --- | --- |
| Mer | 23 | 31.062 | 3.343 | -2.412 |
| FLT3 | 7 | 12.314 | 2.440 | -2.178 |
| FAK1 | 25 | 32.414 | 3.443 | -2.153 |
| Tyk2 | 0 | 2.398 | 1.185 | -2.023 |
| JAK1 | 0 | 2.381 | 1.191 | -1.999 |
| Met | 18 | 24.009 | 3.187 | -1.885 |
| EphB4 | 0 | 1.998 | 1.077 | -1.855 |
| JAK3 | 1 | 3.586 | 1.415 | -1.827 |
| MAP2K7 | 2 | 0.758 | 0.690 | 1.801 |
| MEK1/MAP2K1 | 2 | 0.798 | 0.692 | 1.736 |
| SEK1/MAP2K4 | 2 | 0.792 | 0.701 | 1.724 |
| Axl | 32 | 37.358 | 3.497 | -1.532 |
| FAK2 | 17 | 21.512 | 3.079 | -1.465 |
| Fyn | 7 | 10.348 | 2.351 | -1.424 |
| Lmr1 | 0 | 1.202 | 0.861 | -1.397 |
| Kit | 9 | 12.413 | 2.469 | -1.382 |
| ASK/MAP3K5 | 1 | 0.372 | 0.483 | 1.299 |
| Src | 19 | 23.164 | 3.236 | -1.287 |
| CDK2 | 1 | 0.376 | 0.485 | 1.287 |
| Wee2 | 1 | 0.384 | 0.486 | 1.268 |
| CDC2/CDK1 | 1 | 0.387 | 0.487 | 1.258 |
| ERK2 | 1 | 0.388 | 0.487 | 1.257 |
| RAF1 | 1 | 0.388 | 0.487 | 1.256 |
| FGFR3 | 6 | 8.711 | 2.161 | -1.255 |
| MKK6/MAP2K6 | 1 | 0.390 | 0.488 | 1.250 |

| Oligonucleotides for RT-PCR (Human) | SOURCE | IDENTIFIER |
| --- | --- | --- |
| *RPL41* F: TTGCCCTGTCACTACCTGTG | This paper | N/A |
| *RPL41* R: CAAACCACACCACGTAACCC | This paper | N/A |
| *GPC3* F: ATCCAGCCGAAGAAGGGAAC | This paper | N/A |
| *GPC3* R: TCCATTCCTTGCTGCCTTTTG | This paper | N/A |
| *ABL1* F: GTGGGCTGCAAATCCAAGAA | This paper | N/A |
| *ABL1* R: ATGCTACTGGCCGCTGAAG | This paper | N/A |
| *ABL2* F: GACACTTCACTTTGCTGCCTG | This paper | N/A |
| *ABL2* R: AGTGCCTGGGGTTCAACATC | This paper | N/A |
| *INSR* F: GGGAACTACAGCGTGCGAAT | This paper | N/A |
| *INSR* R: CACGTAGAAATAGGTGGGTTCC | This paper | N/A |
| *SYK* F: AGAGCGAGGAGGAGCGG | This paper | N/A |
| *SYK* R: CGAAAAAGAAGGGCAGGTGG | This paper | N/A |
| *AKT1* F: TCTCCCAGGAGGTTTTTGGG | This paper | N/A |
| *AKT1* R: AAGACAGGACCAGGATGCAG | This paper | N/A |
| *AKT2* F: GCTAGGTGACAGCGTACCAC | This paper | N/A |
| *AKT2* R: TTGTGGAGCCAGCCTTCTTT | This paper | N/A |
| *AKT3* F: GCAGCAGAGAATCCAAACCCT | This paper | N/A |
| *AKT3* R: TCCCCTCTTCTGAACCCAAC | This paper | N/A |
| *GSK3b* F: CAACTGCCCGACTAACACCA | This paper | N/A |
| *GSK3b* R: TGAATCCGAGCATGAGGAGG | This paper | N/A |
| *MAPK1* F: GACTGGACGTGCTCAGACAT | This paper | N/A |
| *MAPK1* R: CCTCCAAACGGCTCAAAGGA | This paper | N/A |
| *DDR1* F: AGAGCGATGAGAGGTGTCTGA | This paper | N/A |
| *DDR1* R: GGCAGCATCTCTTGGCATTC | This paper | N/A |
| *HER4* F: GGACGGGCCATTCCACTTTA | This paper | N/A |
| *HER4* R: AGCCCACCAATTACTCCAGC | This paper | N/A |
| *ZAP70* F: TAACGTCCCCAGACAAACCG | This paper | N/A |
| *ZAP70* R: GAGGTTATCGCGCTTCAGGA | This paper | N/A |
| *SRC* F: GCTTCTGCTGTTGACTGGCT | This paper | N/A |
| *SRC* R: TGAATGGTAGCTCTTGCGGT | This paper | N/A |
| *AURKA* F: AAGACTTGGGTCCTTGGGTC | This paper | N/A |
| *AURKA* R: CCTCGTCCGCCACTGAGATA | This paper | N/A |
| *FRK* F: AGAGGCTAGGAATAATGTCCAAACT | This paper | N/A |
| *FRK* R: ACCCTCTTATCCTACCAGACC | This paper | N/A |
| *YES1* F: TGCGGTAGCAGCGACTCA | This paper | N/A |
| *YES1* R: CCCTTTGCTGAAGATGACGG | This paper | N/A |
| *HCK* F: GCCCAGGATGGGGTGCAT | This paper | N/A |
| *HCK* R: CCTGGTGTGTTGCTGTTGTG | This paper | N/A |

**Supplemental Table 4.** Human Real-Time PCR Primers

| ID | PepProtein_PhosLink | Kinase_Rank |
| --- | --- | --- |
| ZAP70_313_325 | P43403_319 | 0 |
| RB_804_816 | P06400_805 | 0 |
| PTN6_558_570 | P29350_564 | 0 |
| PTN6_531_541 | P29350_536 | 0 |
| PLCG1_764_776 | P19174_771 | 0 |
| PAXI_24_36 | P49023_31 | 0 |
| PAXI_111_123 | P49023_118 | 0 |
| EGFR_1190_1202 | P00533_1197 | 0 |
| DCX_67_79 | O43602_70 | 0 |
| CTNB1_79_91 | P35222_86 | 0 |
| CDK2_8_20 | Q00535_15 | 0 |
| CBL_693_705 | P22681_700 | 0 |
| ANXA1_14_26 | P04083_21 | 0 |
| ZBT16_621_633 | P17030_202 | 1 |
| PTN11_57_67 | P29350_61 | 1 |
| CD3Z_105_117 | P20963_111 | 1 |
| NTRK2_696_708 | P30530_703 | 2 |
| EFS_246_258 | O43281_253 | 2 |
| PTN11_580_590 | Q06124_584 | 3 |
| PDPK1_369_381 | O15530_376 | 3 |
| GAB1_622_632 | Q13480_627 | 3 |
| EGFR_1165_1177 | P00533_1172 | 3 |
| PLCG1_777_789 | P19174_783 | 4 |
| PGFRB_771_783 | Q9Y6L7_954 | 4 |
| PGFRB_768_780 | P09619_775 | 4 |
| FRK_380_392 | P42685_387 | 4 |
| EPHA7_607_619 | P29317_594 | 4 |
| EGFR_1103_1115 | P00533_1110 | 4 |
| CD79A_181_193 | P11912_182 | 4 |
| CD28_203_215 | P10747_209 | 4 |
| 41_654_666 | P11171_660 | 4 |
| VGFR1_1040_1052 | P17948_1048 | 5 |
| MET_1227_1239 | P08581_1230 | 5 |
| GAB2_638_648 | Q8WWW8_560 | 5 |
| EPHA2_765_777 | P29322_793 | 5 |
| CDK4_11_23 | P11802_21 | 5 |
| GSK3B_210_222_C218S | P49840_279 | 6 |
| EPHA1_774_786 | P21709_781 | 6 |
| AKT1_309_321_C310S | P51812_234 | 6 |
| ZAP70_485_497 | P43403_493 | 7 |
| PECA1_706_718 | P16284_713 | 7 |
| BTLA_252_262 | Q7Z6A9_257 | 7 |
| DYR1A_312_324 | Q13627_321 | 8 |
| PGFRB_572_584 | P16234_574 | 9 |
| LYN_391_403 | P51451_389 | 9 |
| TEC_512_524 | Q08881_512 | 10 |
| LYN_501_512 | P07948_508 | 10 |
| MBP_259_271 | P02686_261 | 11 |
| EPHB1_771_783 | P29323_780 | 11 |
| PLCG1_1246_1258 | P19174_1253 | 12 |
| PGFRB_1014_1028 | P09619_1021 | 12 |
| PGFRB_1002_1014 | P09619_1009 | 12 |
| MAPK3_198_210_C203S | Q16644_204 | 12 |
| KIT_930_942_C942S | P10721_936 | 12 |
| IRS2_626_638 | Q9Y4H2_628 | 12 |
| INSR_992_1004 | P08069_973 | 12 |

**Supplemental Table 5.** ABL1 Substrates and Ranking


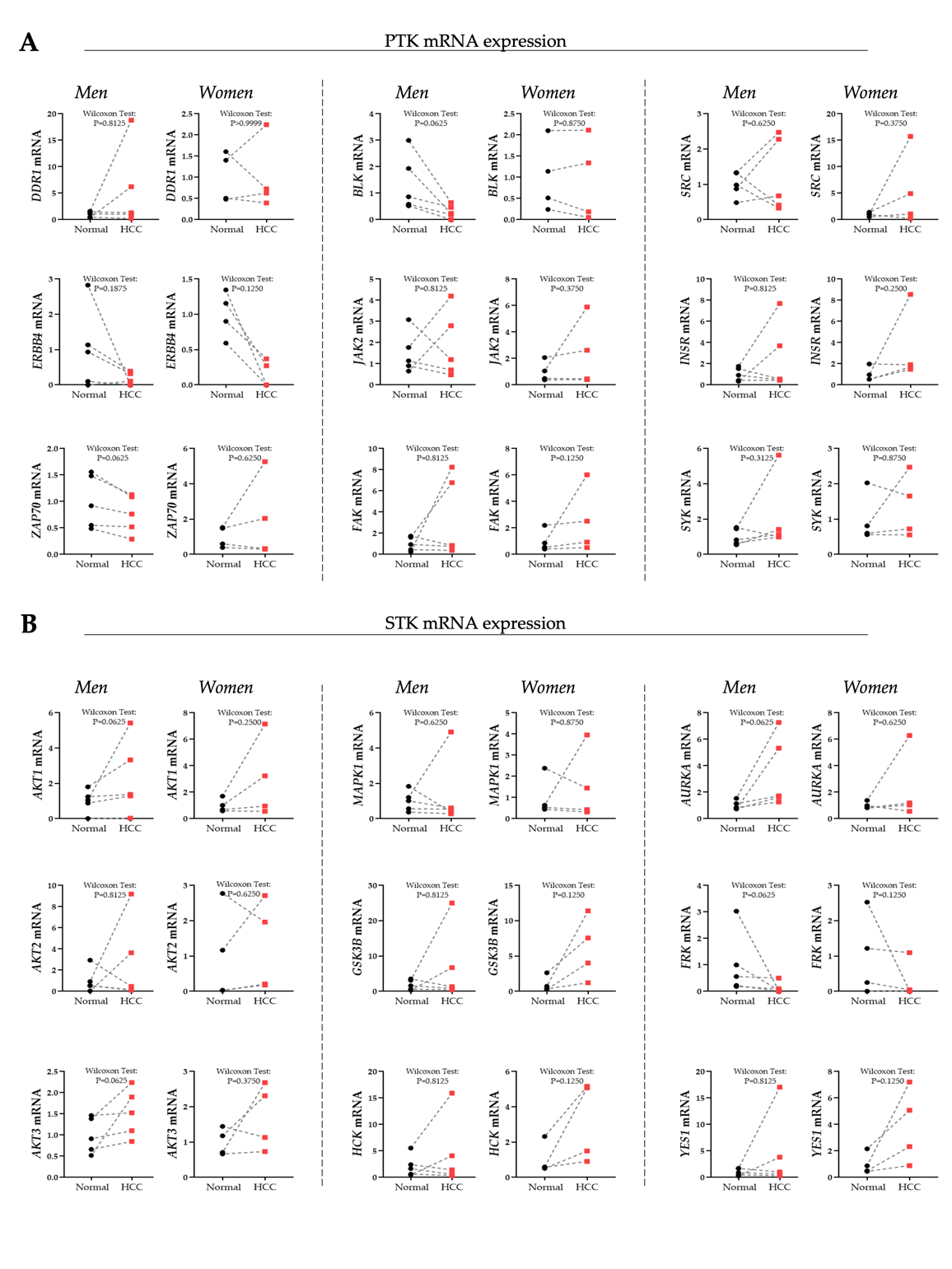
**SUPPLEMENTAL FIGURES**

**Figure S1. RT-PCR validation of altered kinases**

1. RT-PCR validation of PTK kinases identified in the kinome analysis was quantified in a sample of n=9, comprising n=5 males and n=4 females as individual biological replicates. These evaluations were performed on non-cancerous tissues (indicated by black circles) and tumor tissues (indicated by red squares).
2. RT-PCR validation of STK kinases identified in the kinome analysis was quantified in a sample of n=9, comprising n=5 males and n=4 females as individual biological replicates. These evaluations were performed on non-cancerous tissues (indicated by black circles) and tumor tissues (indicated by red squares).


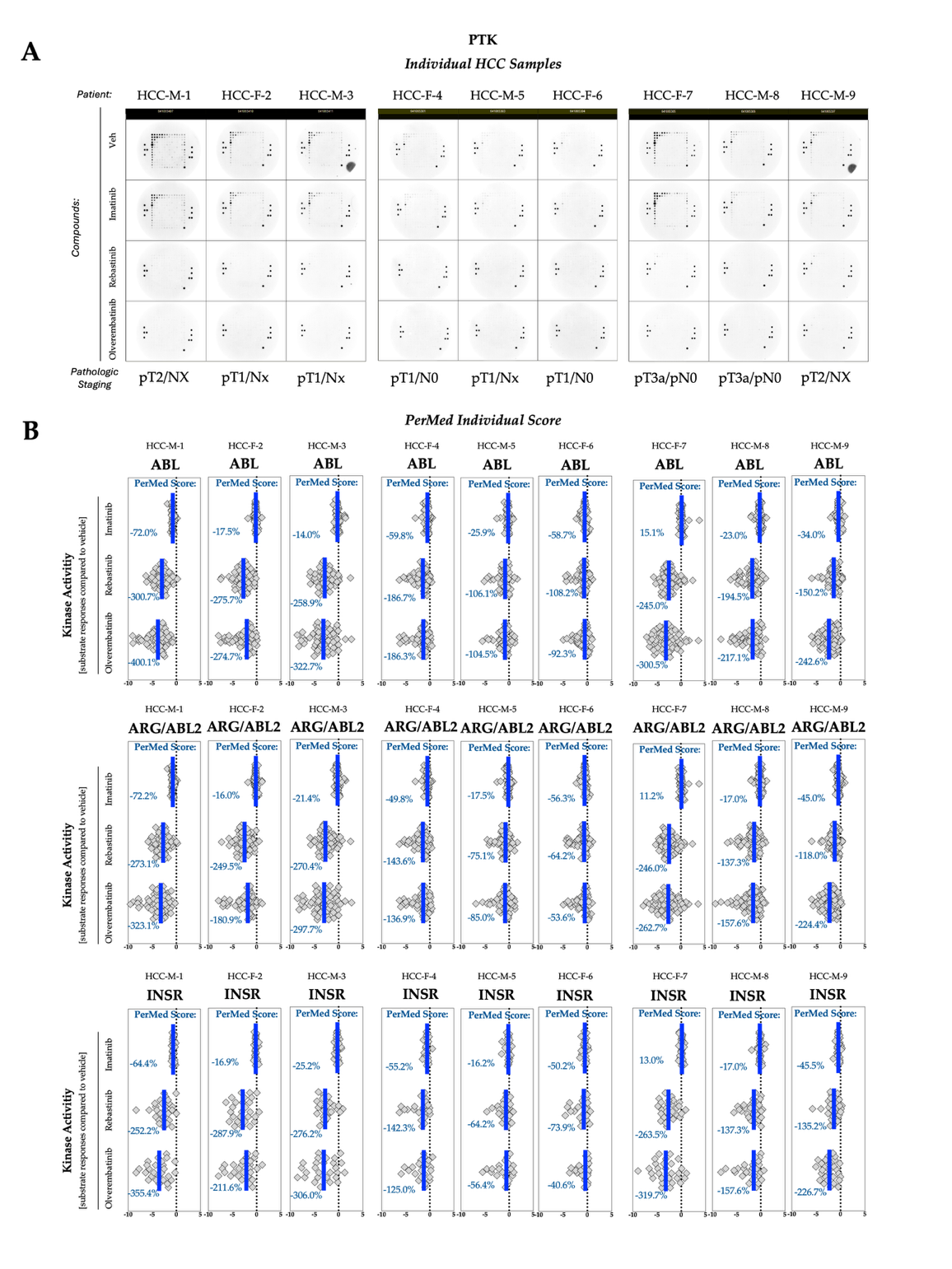


**Figure S2. The efficacy of ABL inhibitors in nine HCC tumors.**

1. Images show phosphorylated PTK PamChip during the final cycle for individual biological replicates of male and female patients’ HCC tumors treated with vehicle and ABL inhibitors. The tumor pathological staging is denoted below each image.
2. The kinase activities of ABL, ARG/ABL2, and INSR, along with the PerMed Score, reflect the percentage change in kinase activity within each individual biological replicate treated with ABL inhibitors relative to vehicle. The blue line represents the mean signal intensity for each substrate relative to the control, with the percentage change indicated above.


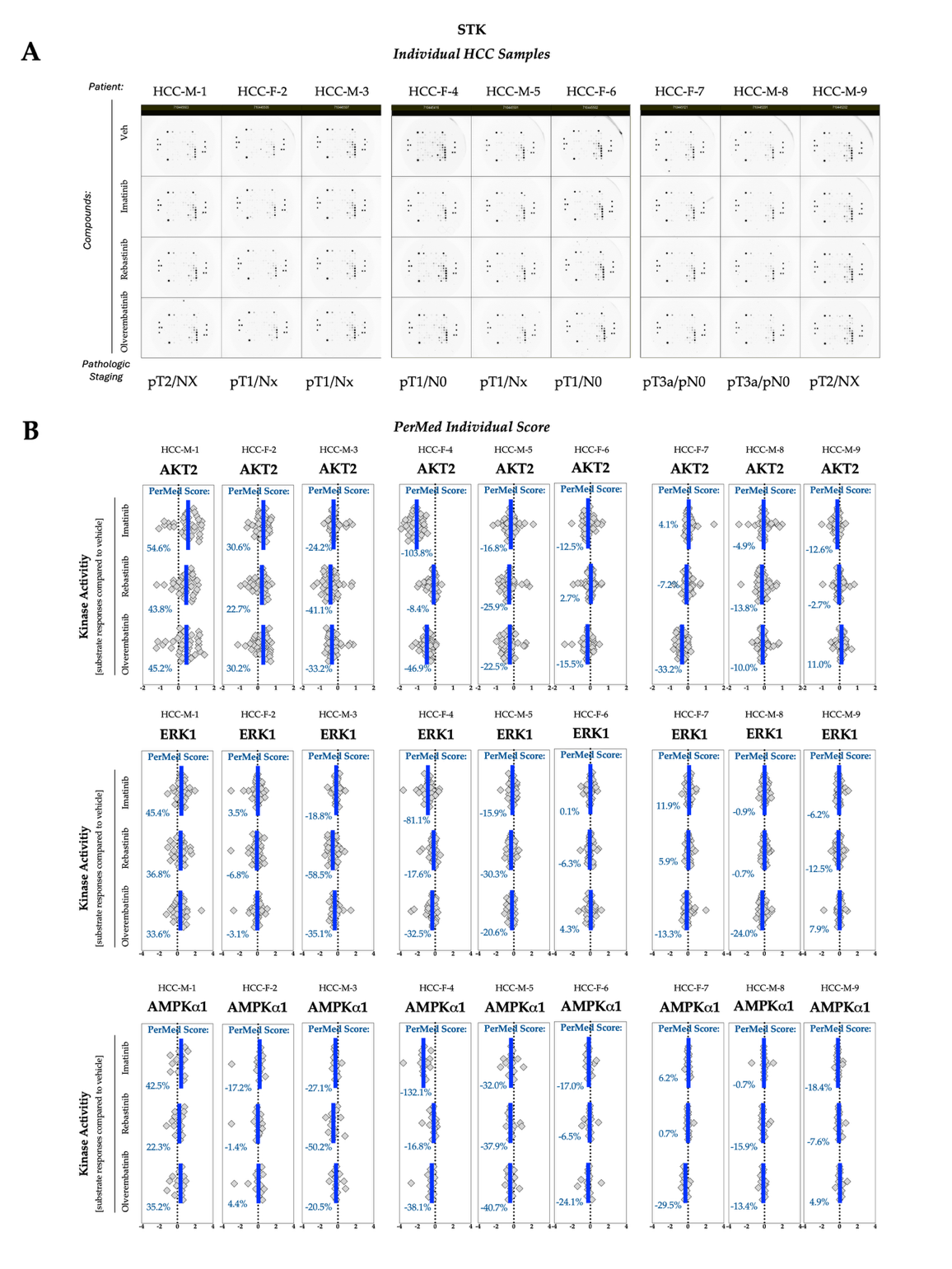


**Figure S3. Off-target effects on STK kinases of ABL inhibitors in nine HCC tumors.**

1. Images show phosphorylated STK PamChip during the final cycle for individual biological replicates of male and female patients’ HCC tumors treated with vehicle and ABL inhibitors. The tumor pathological staging is denoted below each image.
2. The kinase activities of AKT2, ERK1, and AMPKα1, along with the PerMed Score, reflect the percentage change in kinase activity within each individual biological replicate treated with ABL inhibitors relative to vehicle. The blue line represents the mean signal intensity for each substrate relative to the control, with the percentage change indicated above.


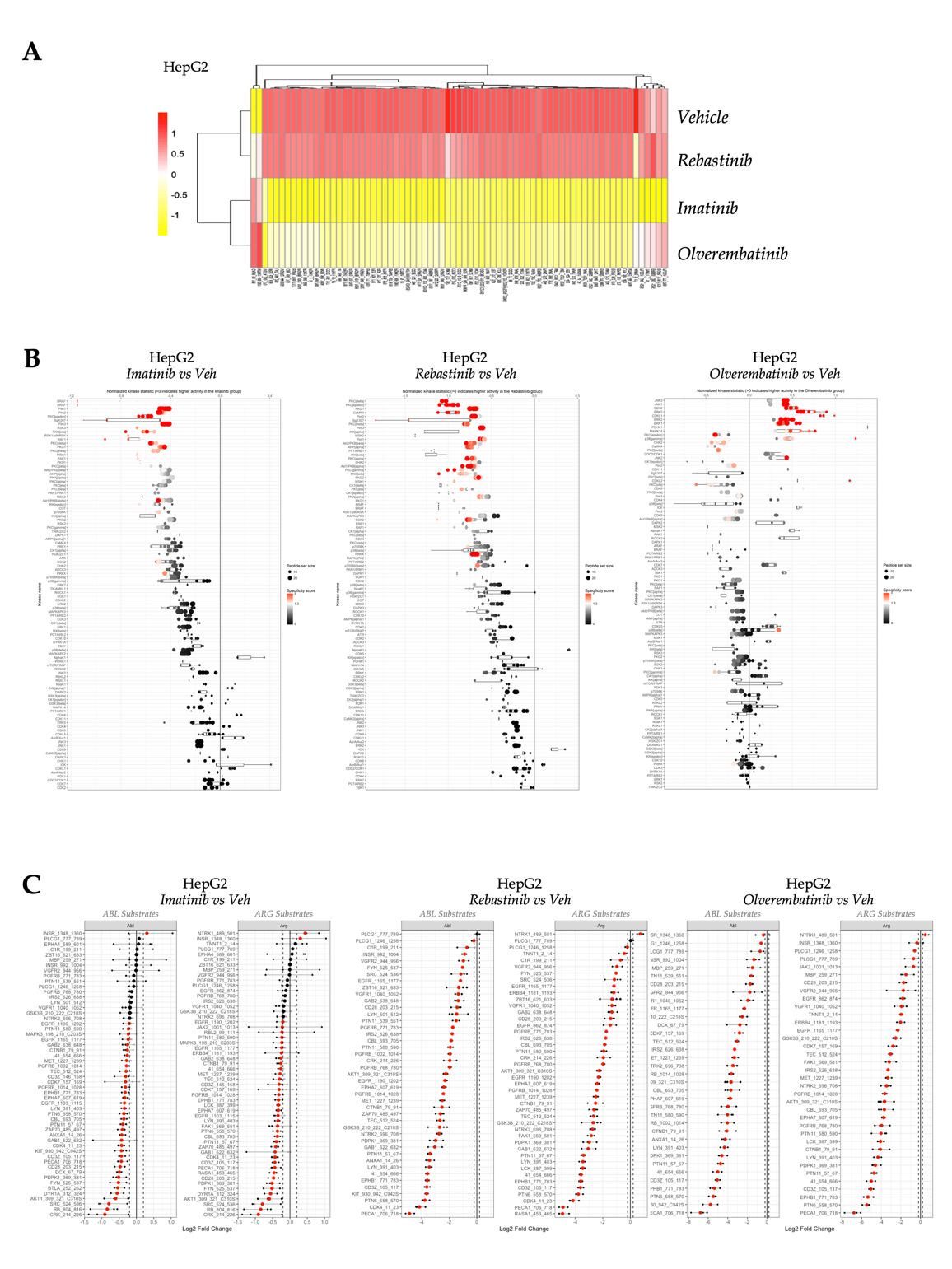


**Figure S4. ABL inhibitors differentially suppress PTK and STK kinases in HepG2**

1. Heatmap showing the phosphorylation of 196 PTK substrates in HepG2 with vehicle, Imatinib, Rebastinib, or Olverembatinib. [n=3, ran as biological replicates and validated individually].
2. Waterfall plot of the STK Upstream Kinase Analysis (UKA), depicting kinase activity in HepG2 comparing Imatinib (left), Rebastinib (middle), and Olverembatinib (right) to vehicle.
3. Waterfall plots of ABL and ARG substrates in HepG2 with Imatinib (left), Rebastinib (middle), and Olverembatinib (right) compared to vehicle. Red points signify substrates passing a logFC threshold of 0.2.


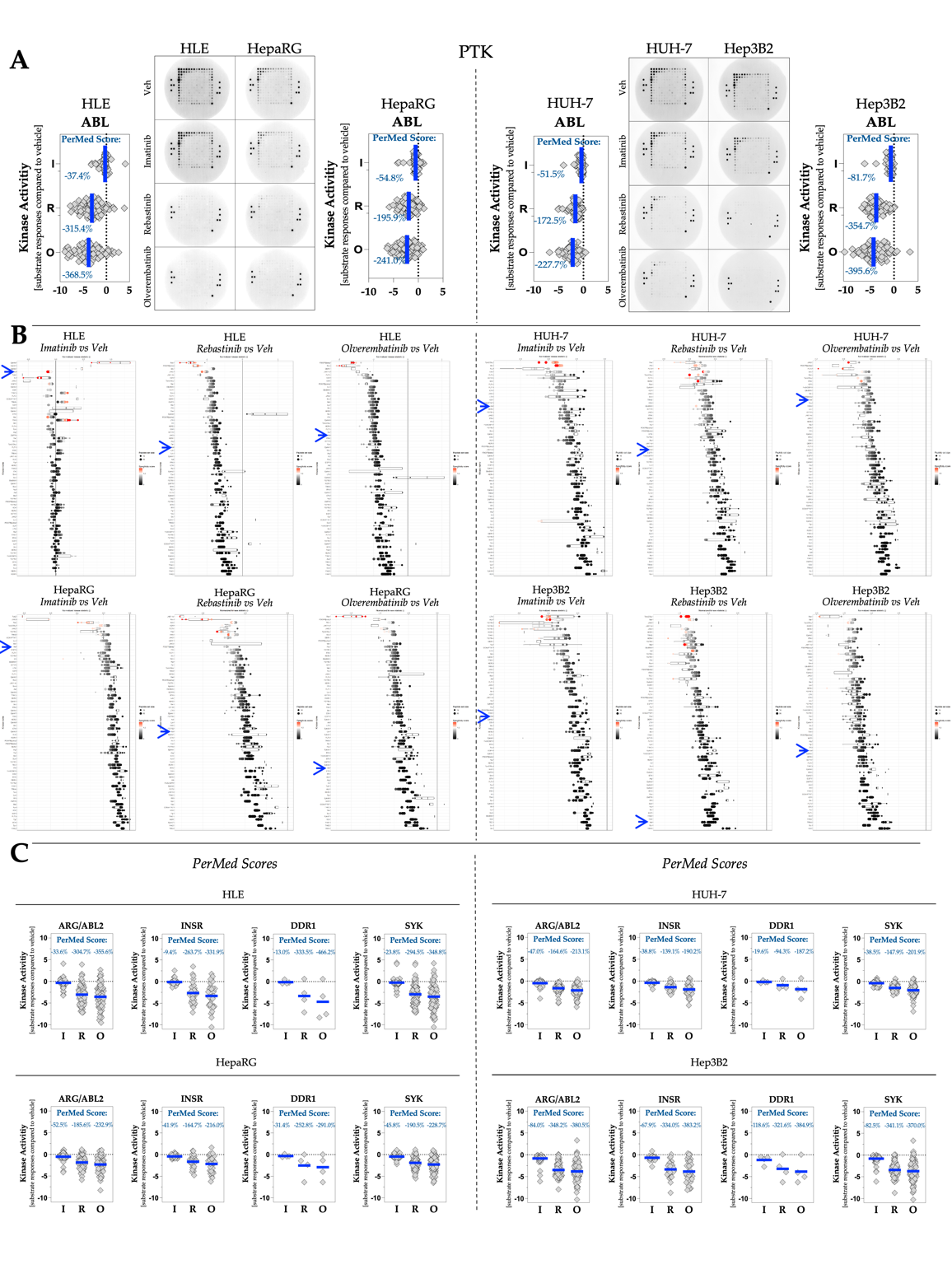


**Figure S5. ABL inhibitors suppress PTK kinases in human hepatocyte cancer cell lines.**

1. Images of phosphorylated PTK PamChip during the final cycle in HLE, HepaRG, Huh7, and Hep3B2 cells. The kinase activity of ABL in the four cell lines. The PerMed Score represents the percentage change in kinase activity in each cell line subjected to ABL inhibitors relative to the vehicle control. The blue line shows the average signal intensity of each substrate relative to the control.
2. Waterfall plot of the PTK Upstream Kinase Analysis (UKA), illustrates kinase activity in HLE, HepaRG, Huh7, and Hep3B2 cells comparing Imatinib (left), Rebastinib (middle), and Olverembatinib (right) to vehicle. Blue arrow denotes the location of ABL.
3. The kinase activity of ARG/ABL2, INSR, DDR1, and SYK in HLE, HepaRG, Huh7, and Hep3B2 cells. The PerMed Score represents the percentage change in kinase activity in each cell line treated with ABL inhibitors relative to the vehicle control. The blue line shows the average signal intensity of each substrate relative to the control.


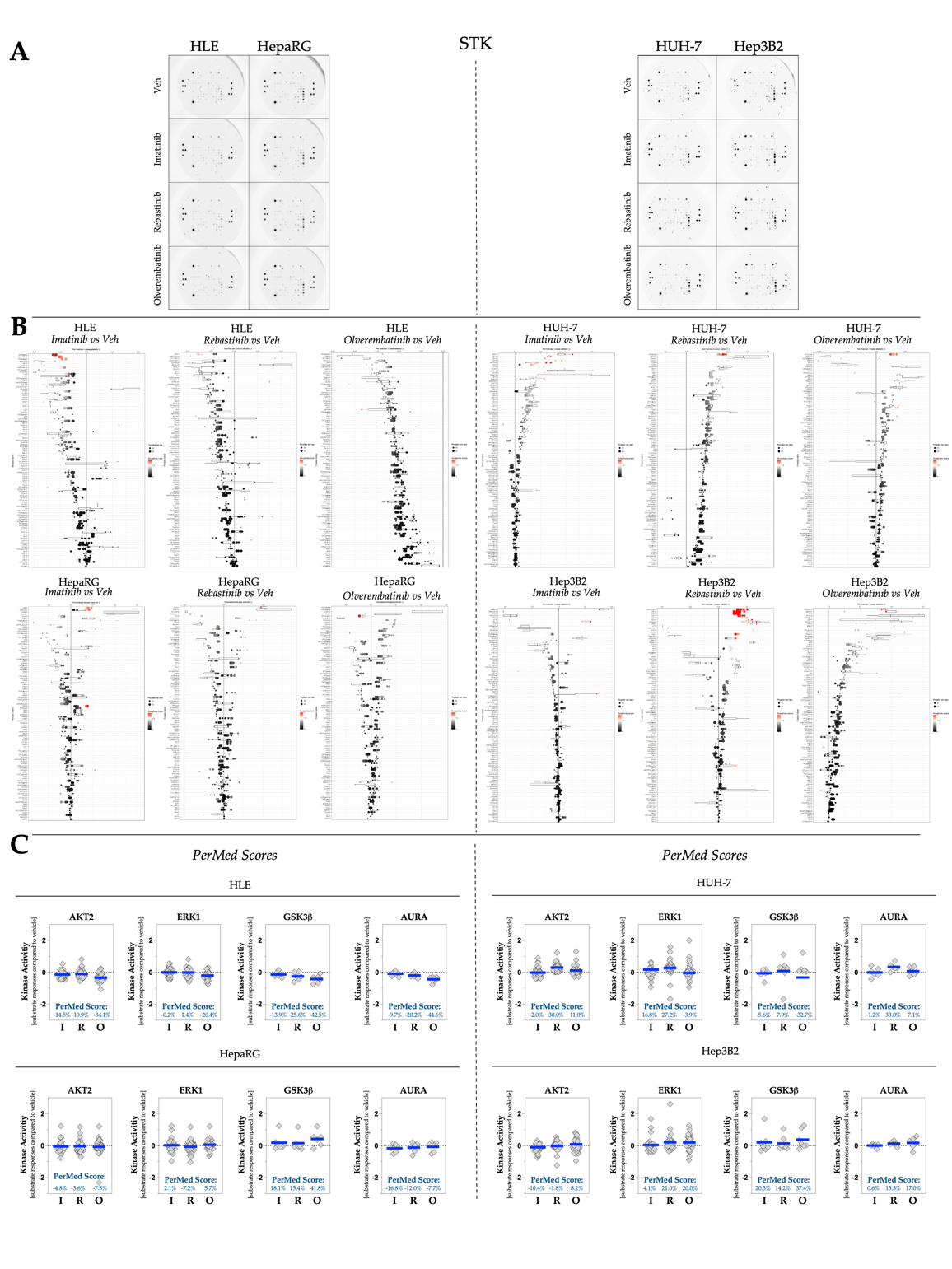


**Figure S6. ABL inhibitors differentially suppress STK kinases in human hepatocyte cancer cell lines.**

1. Images of phosphorylated STK PamChip during the final cycle in HLE, HepaRG, Huh7, and Hep3B2 cells.
2. Waterfall plot of the STK Upstream Kinase Analysis (UKA) presents kinase activity in HLE, HepaRG, Huh7, and Hep3B2 cells comparing Imatinib (left), Rebastinib (middle), and Olverembatinib (right) to vehicle for each cell line.
3. The kinase activity of AKT2, ERK1, GSK3β, and AURA in HLE, HepaRG, Huh7, and Hep3B2 cells. The PerMed Score denotes the percentage change in kinase activity in each cell line subjected to ABL inhibitors relative to the vehicle control. The blue line shows the average signal intensity of each substrate relative to the control.
