# Supplementary material for: Kinase signaling in liver disease via clinical-trial-on-a-PamChip: A distinctive methodology for drug mechanisms and personalized medicine
Source: J Biol Chem. 2026 Mar 18;302(5):111379. doi: 10.1016/j.jbc.2026.111379 (PMC13091354; doi:10.1016/j.jbc.2026.111379)
Supplement: Supplementary Material 2 [file mmc2.pdf]

Appendix A: Individual HCC Samples with ABL Inhibitors

PTK PamChip runs

HCC-M-1

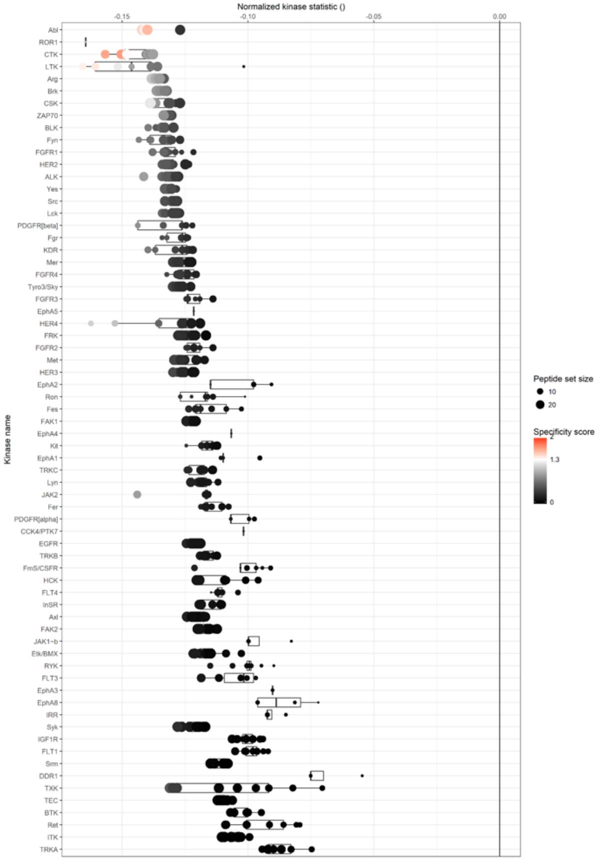

Imatinib vs Veh

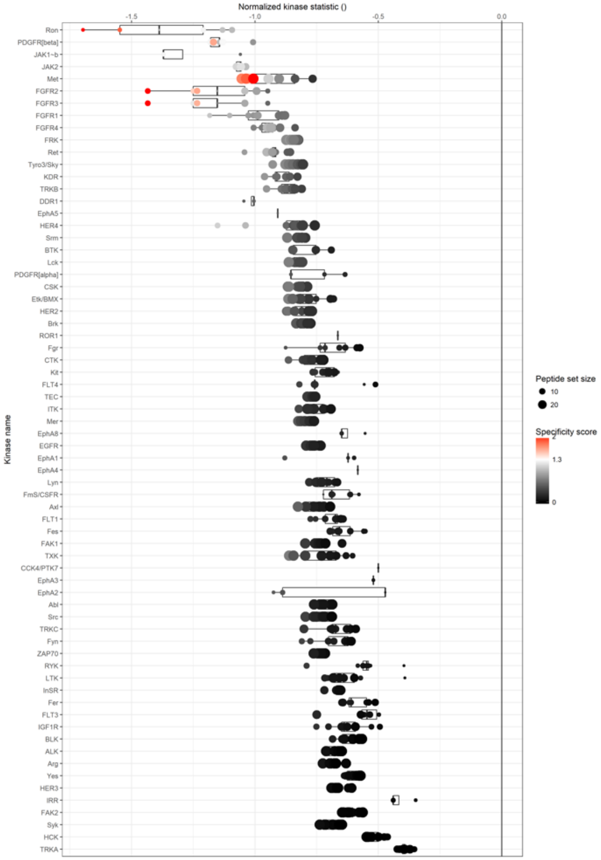

Rebastinib vs Veh

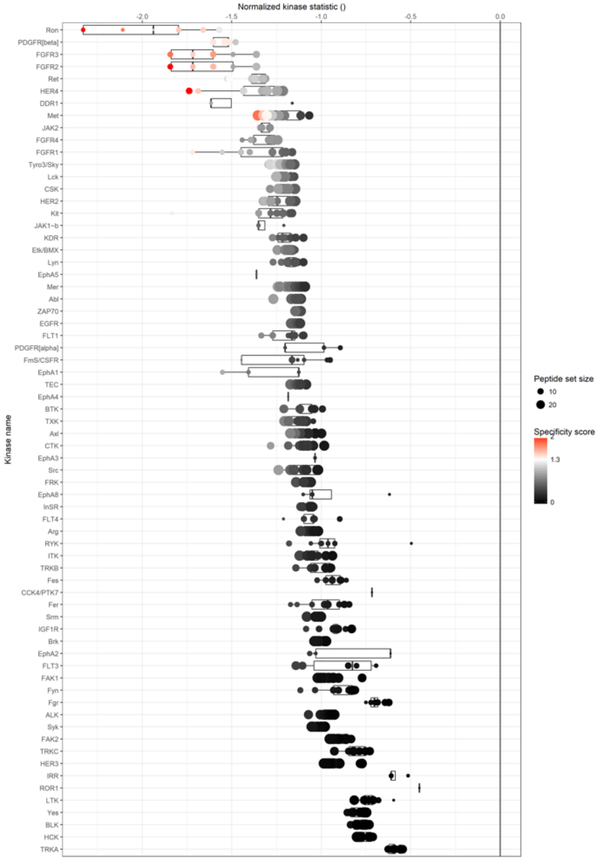

Olverembatinib vs Veh

HCC-F-2

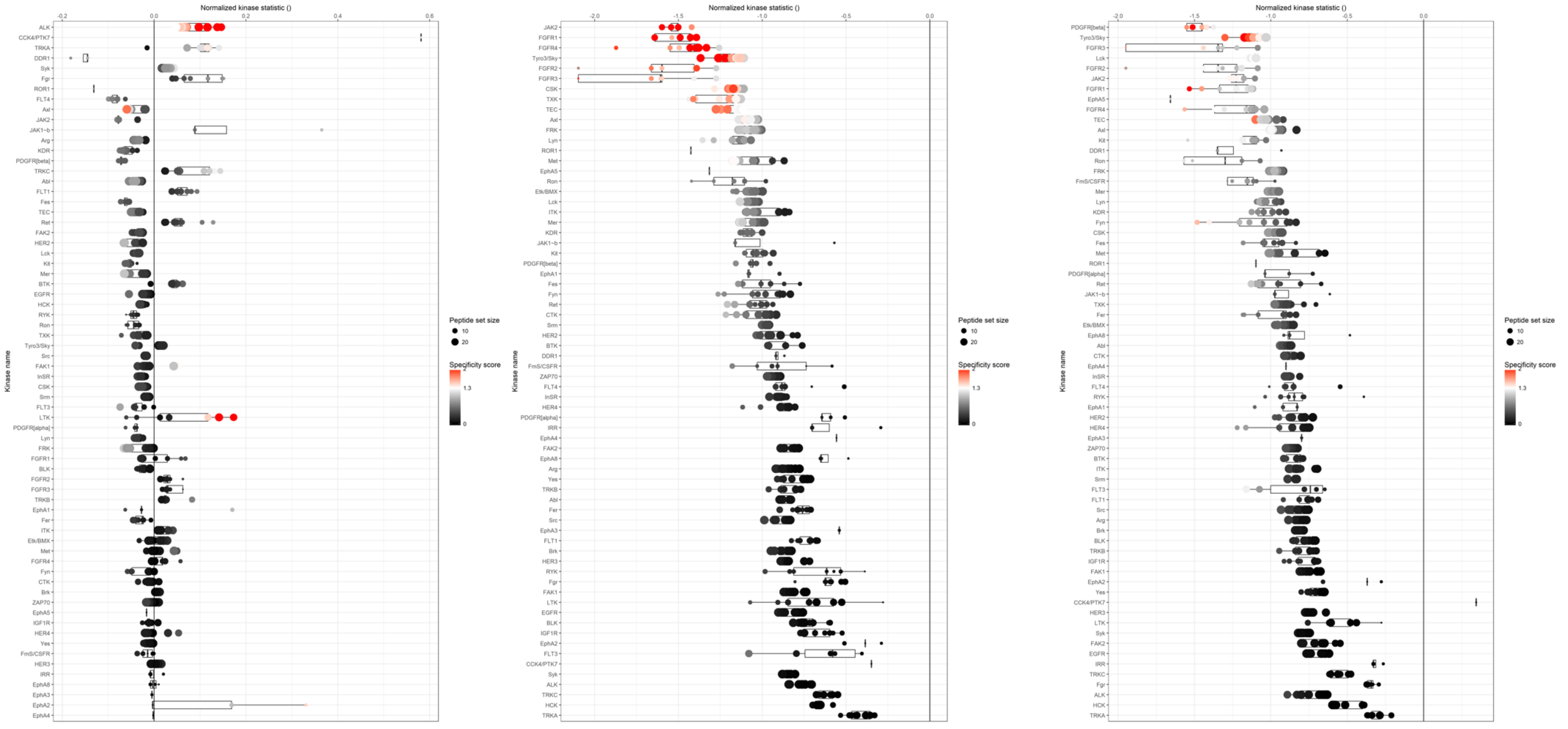

Imatinib vs Veh

Rebastinib vs Veh

Olverembatinib vs Veh

HCC-M-3

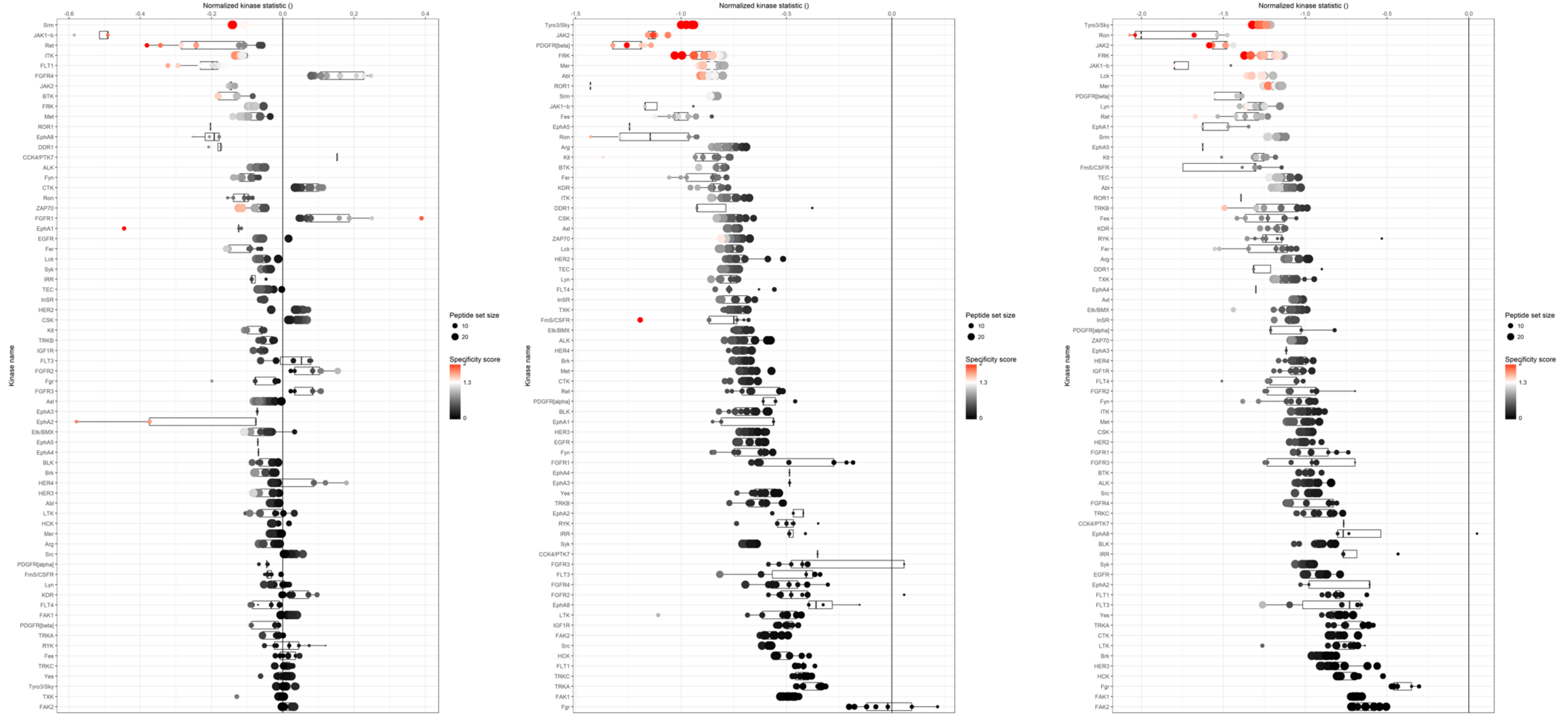

Imatinib vs Veh

Rebastinib vs Veh

Olverembatinib vs Veh

HCC-F-4

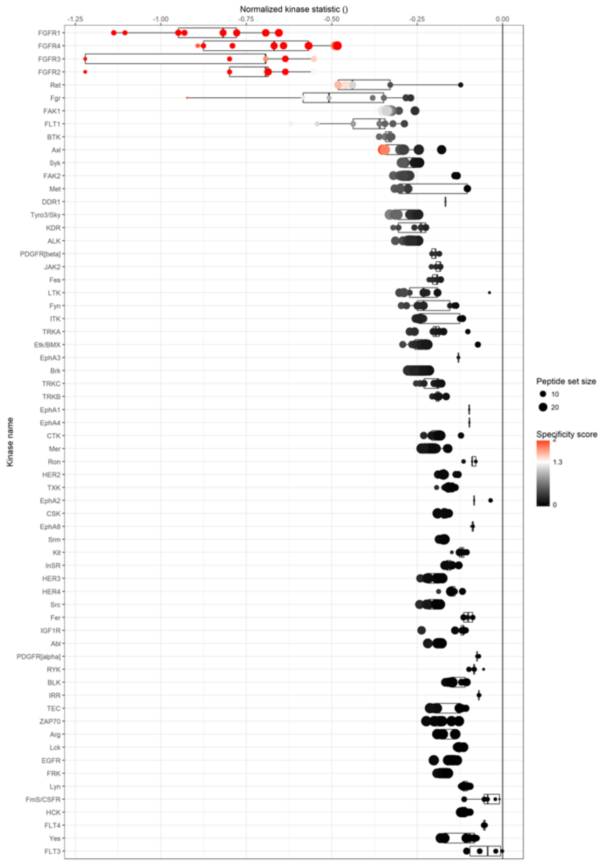

Imatinib vs Veh

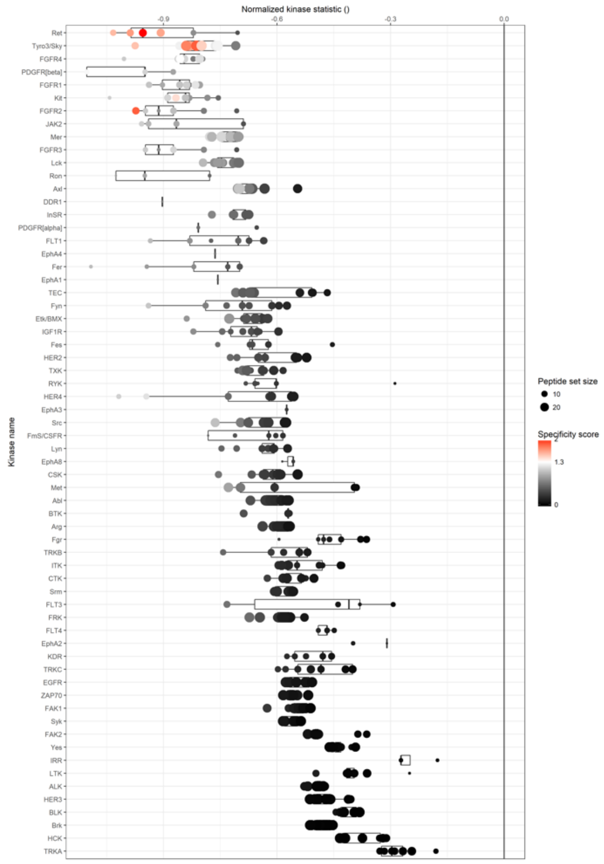

Rebastinib vs Veh

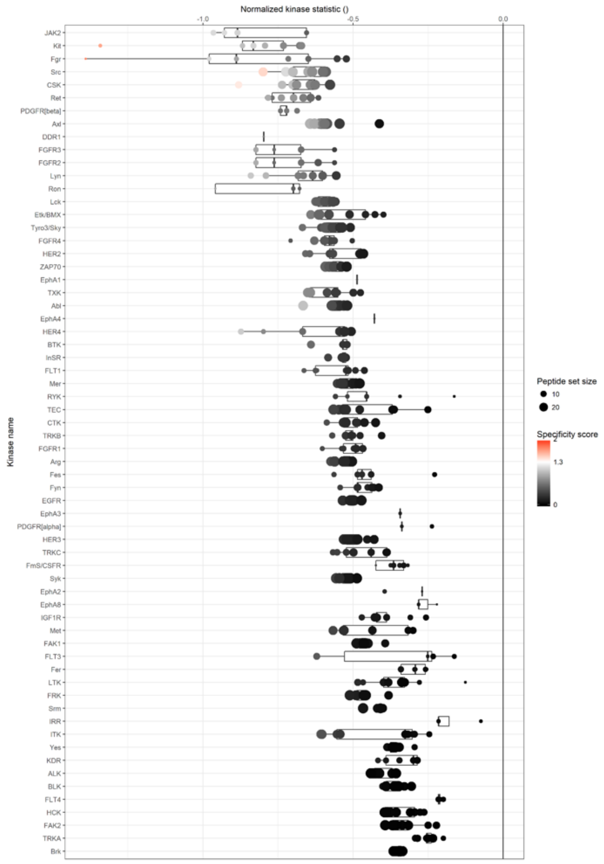

Olverembatinib vs Veh

HCC-M-5

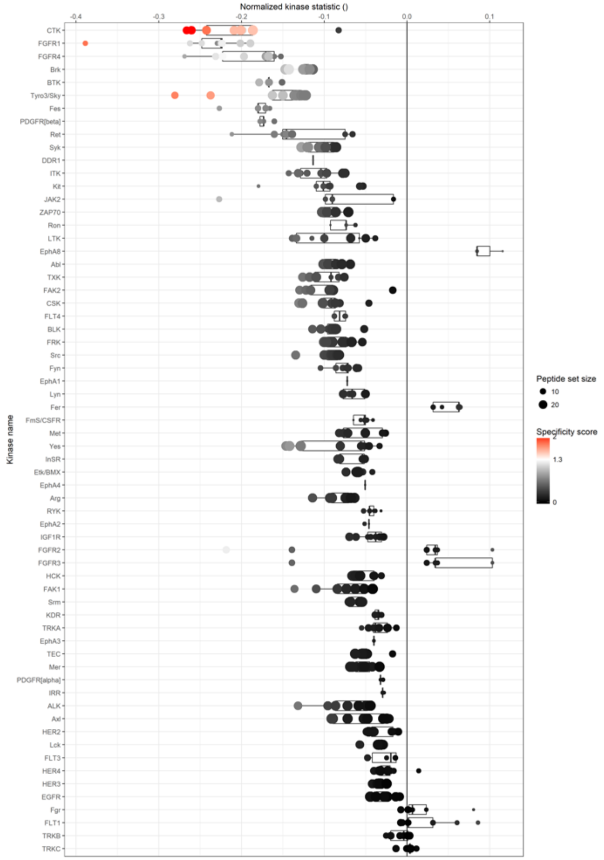

Imatinib vs Veh

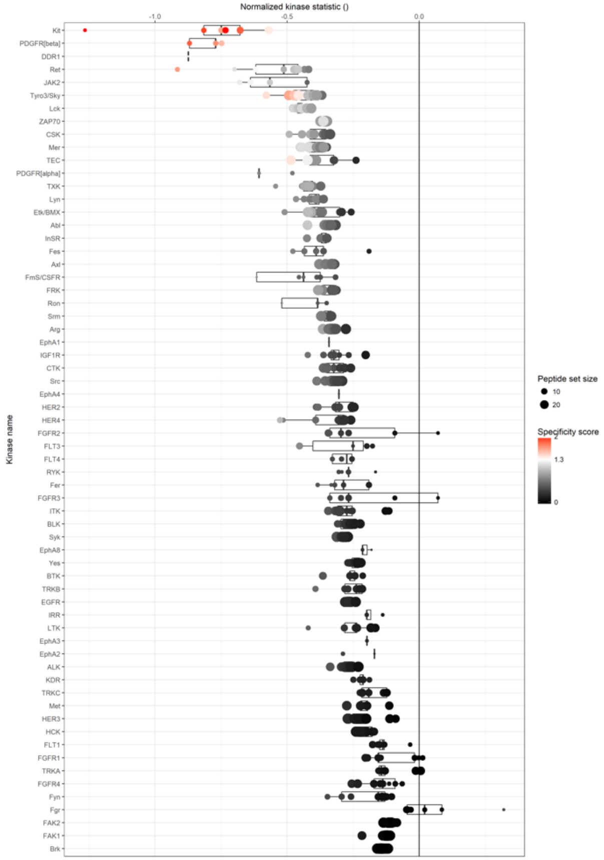

Rebastinib vs Veh

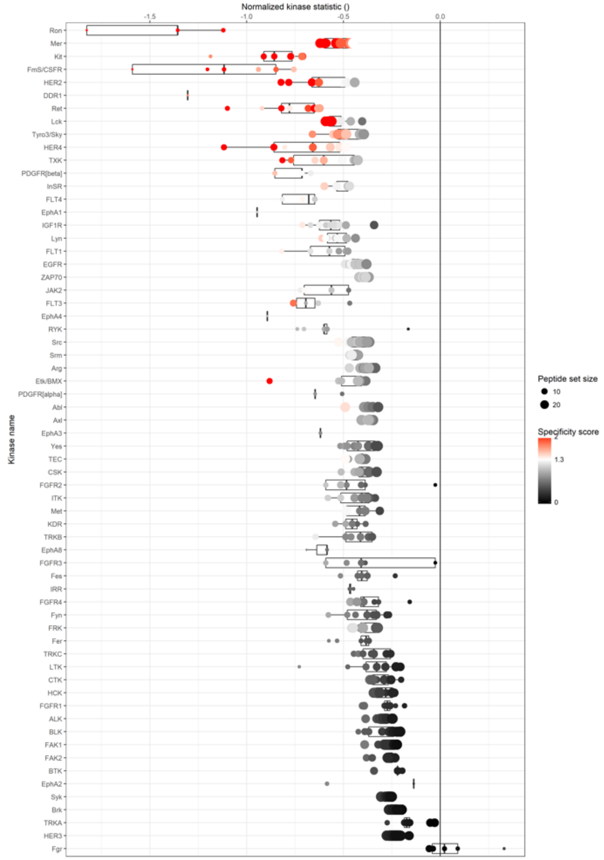

Olverembatinib vs Veh

HCC-F-6

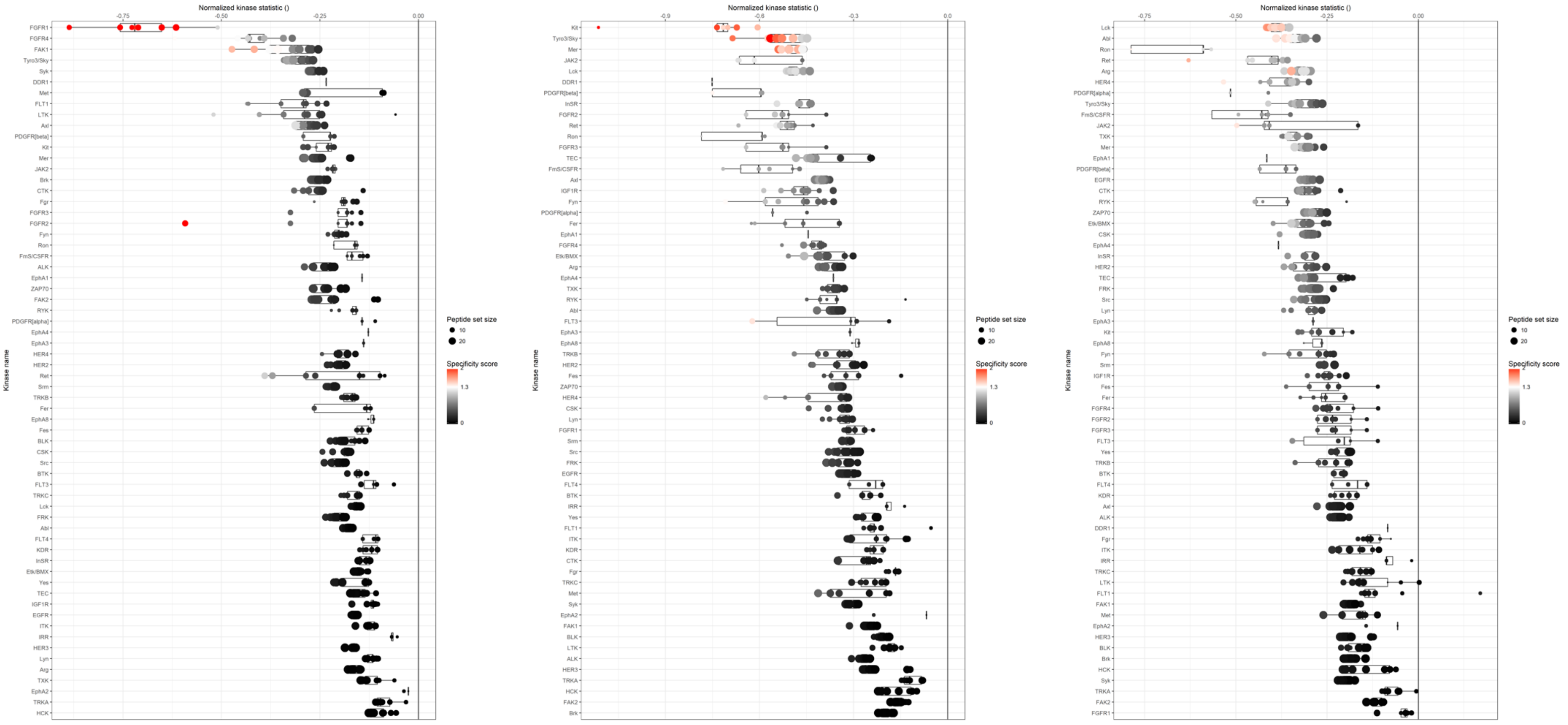

Imatinib vs Veh

Rebastinib vs Veh

Olverembatinib vs Veh

HCC-F-7

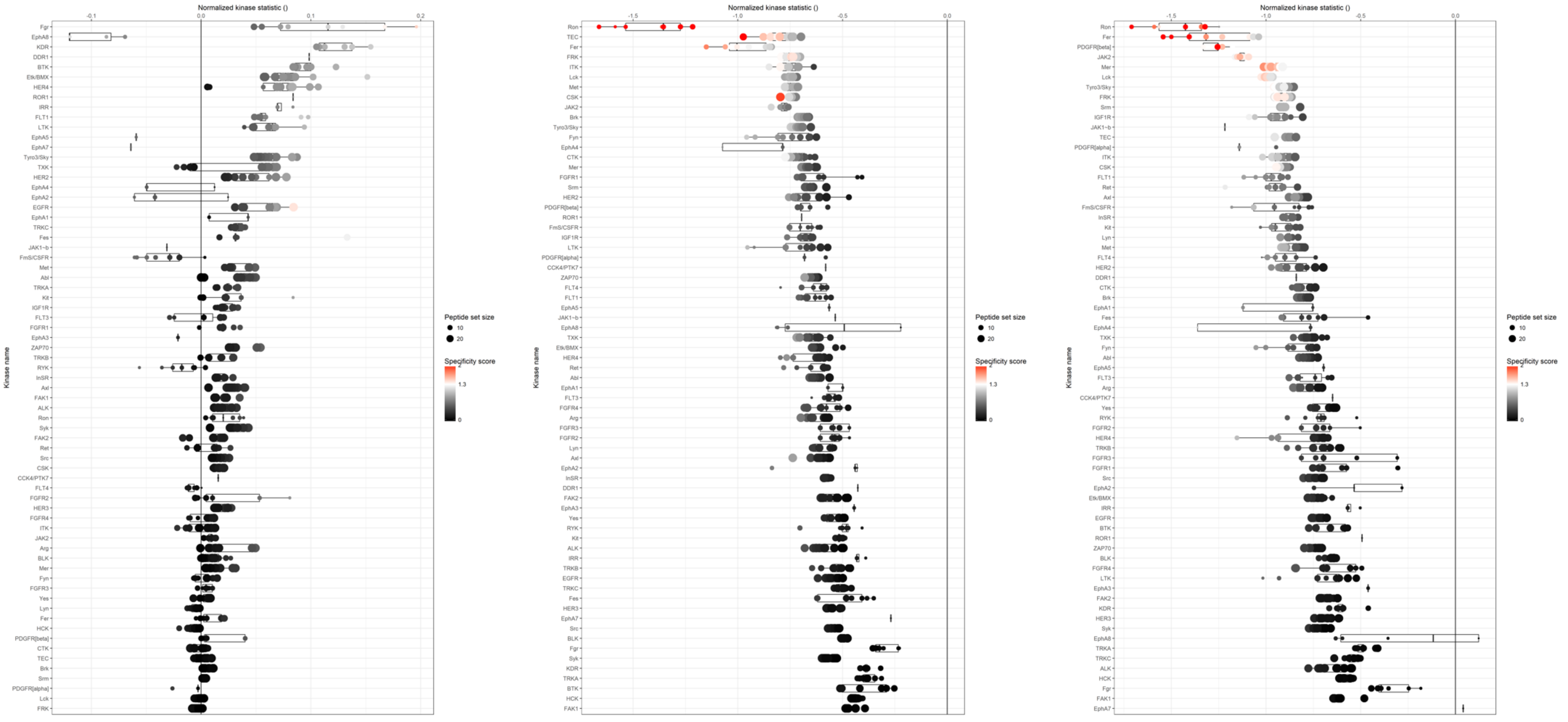

Imatinib vs Veh

Rebastinib vs Veh

Olverembatinib vs Veh

HCC-M-8

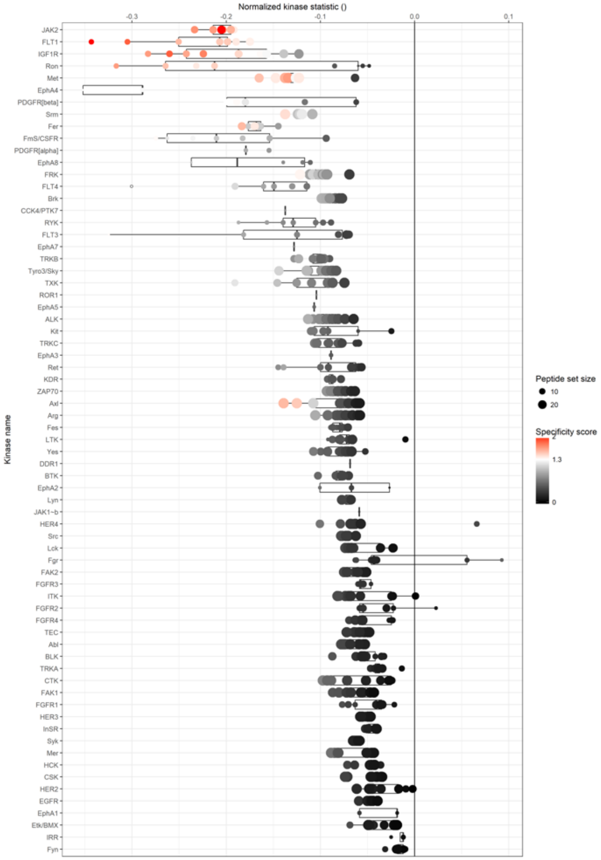

Imatinib vs Veh

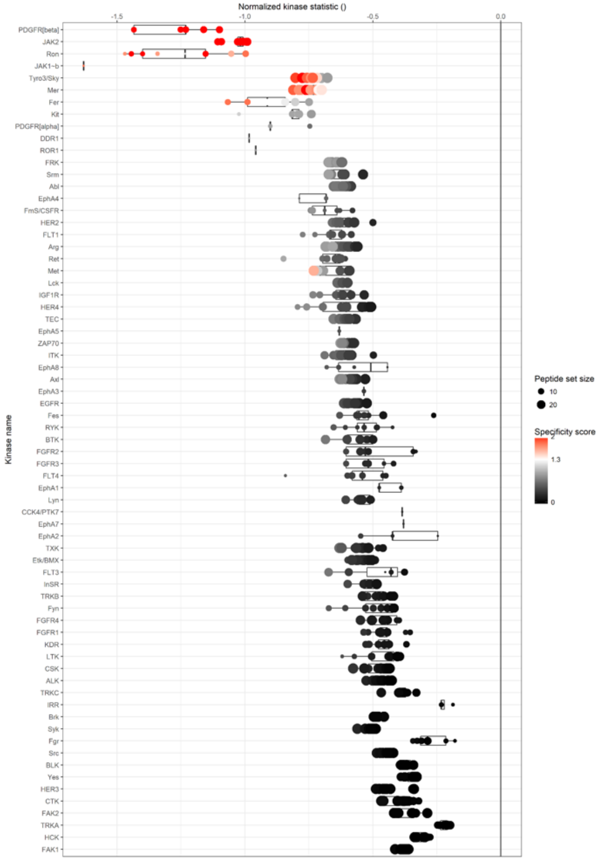

Rebastinib vs Veh

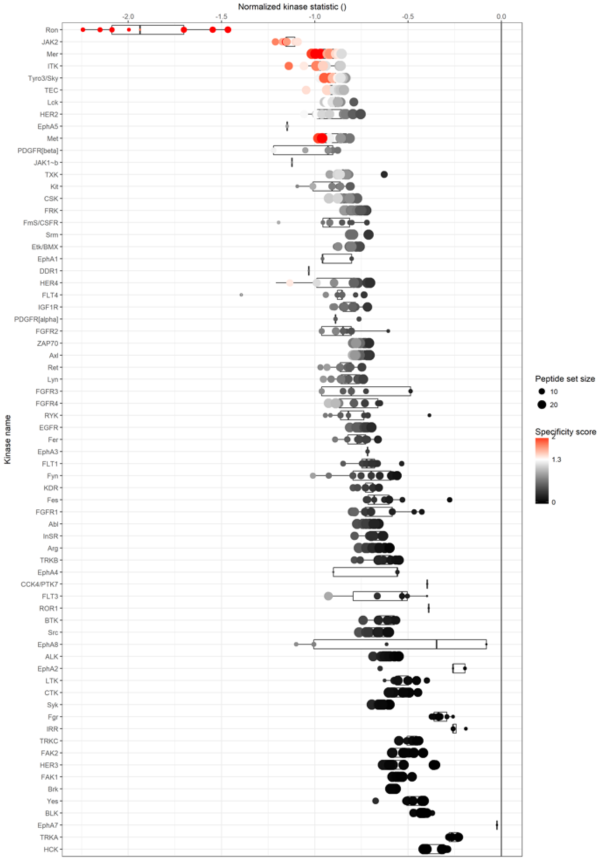

Olverembatinib vs Veh

HCC-M-9

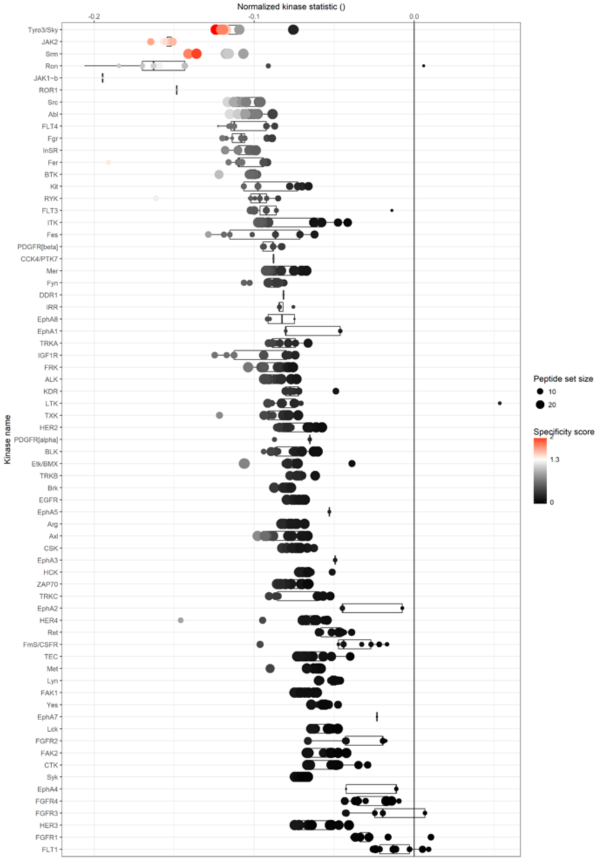

Imatinib vs Veh

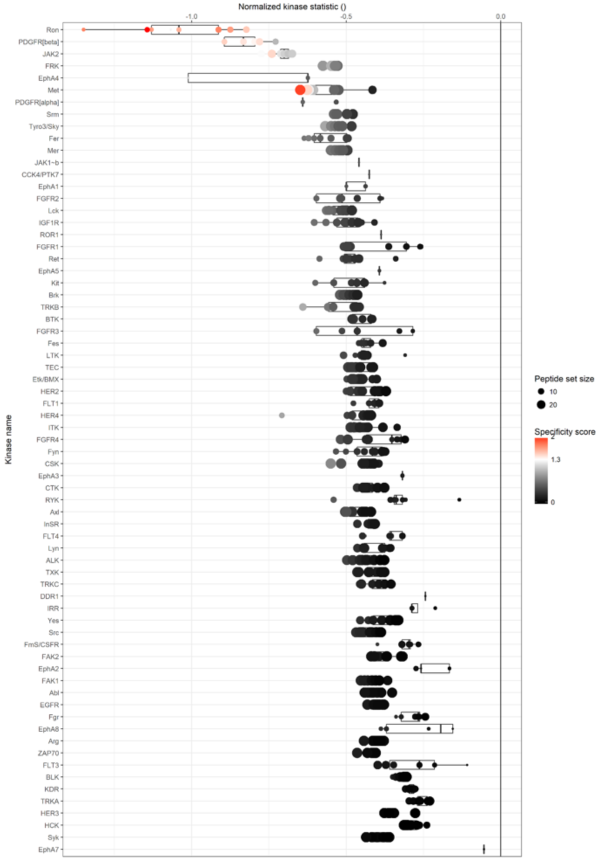

Rebastinib vs Veh

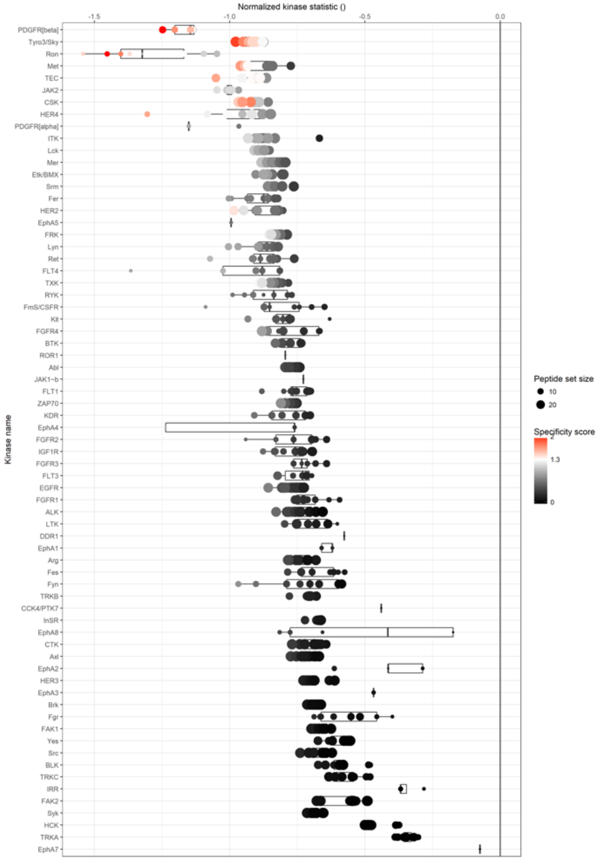

Olverembatinib vs Veh
