# Supplementary material for: Kinase signaling in liver disease via clinical-trial-on-a-PamChip: A distinctive methodology for drug mechanisms and personalized medicine
Source: J Biol Chem. 2026 Mar 18;302(5):111379. doi: 10.1016/j.jbc.2026.111379 (PMC13091354; doi:10.1016/j.jbc.2026.111379)
Supplement: Supplementary Material 3 [file mmc3.pdf]

# Appendix B: Individual HCC Samples with ABL Inhibitors

STK PamChip runs

HCC-M-1

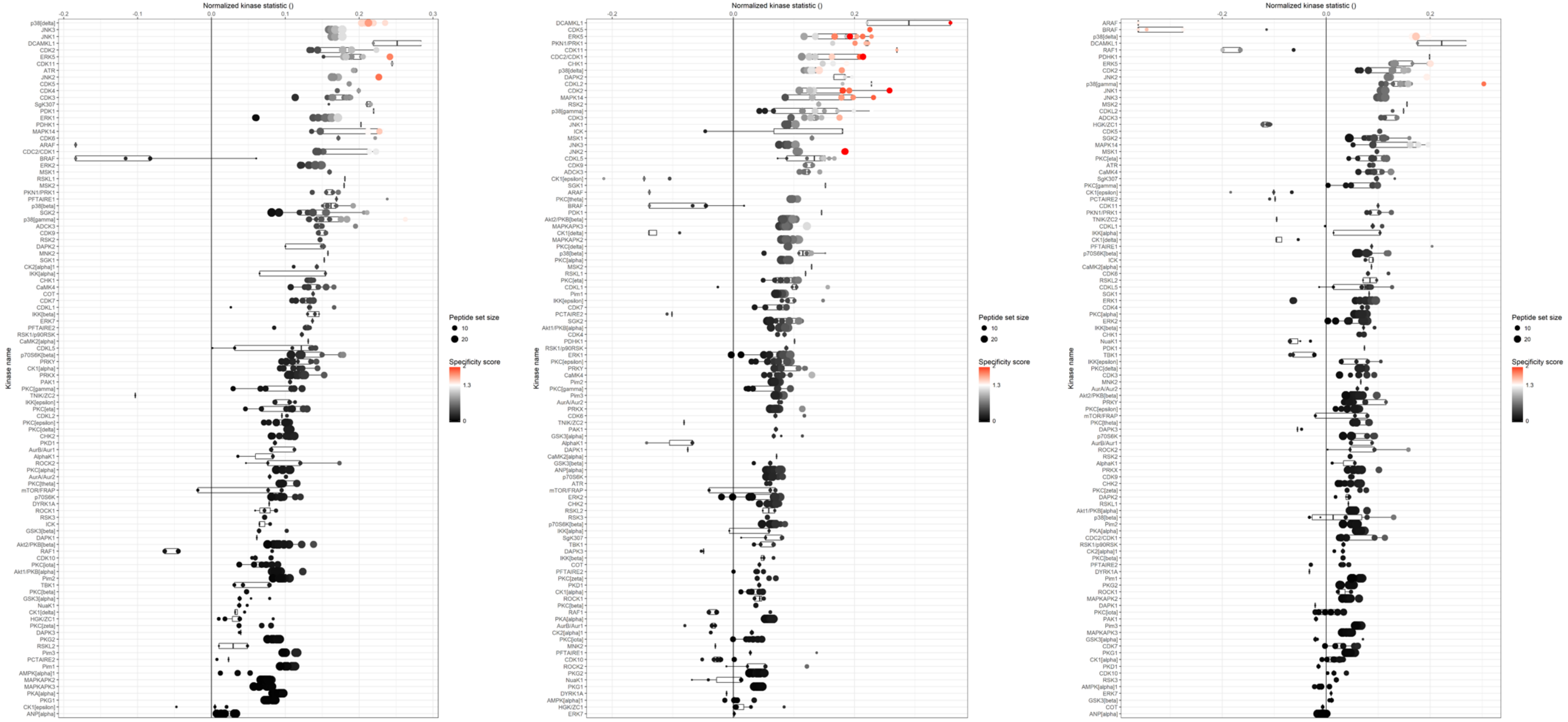


Imatinib vs Veh

Rebastinib vs Veh

Olverembatinib vs Veh

# HCC-F-2

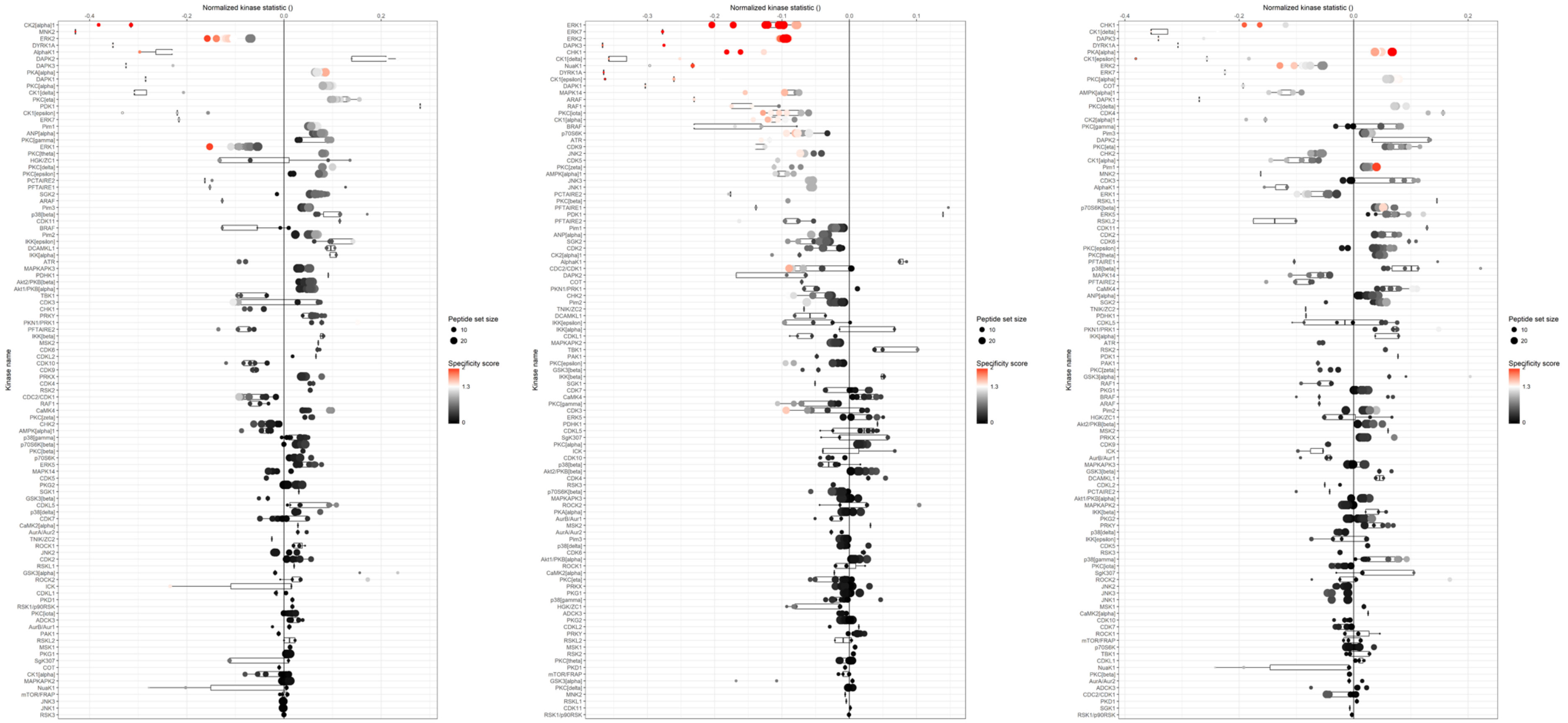


Imatinib vs Veh

Rebastinib vs Veh

Olverembatinib vs Veh

# HCC-M-3

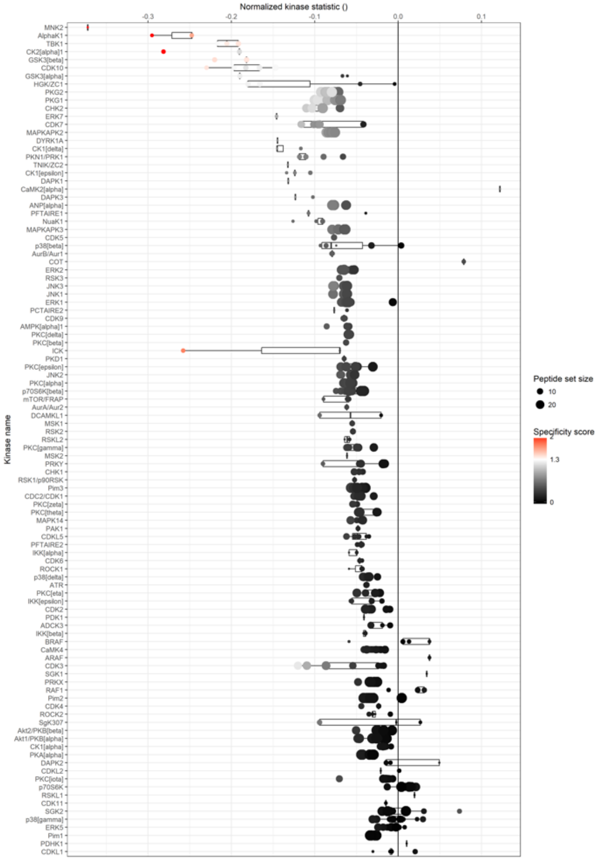


Imatinib vs Veh

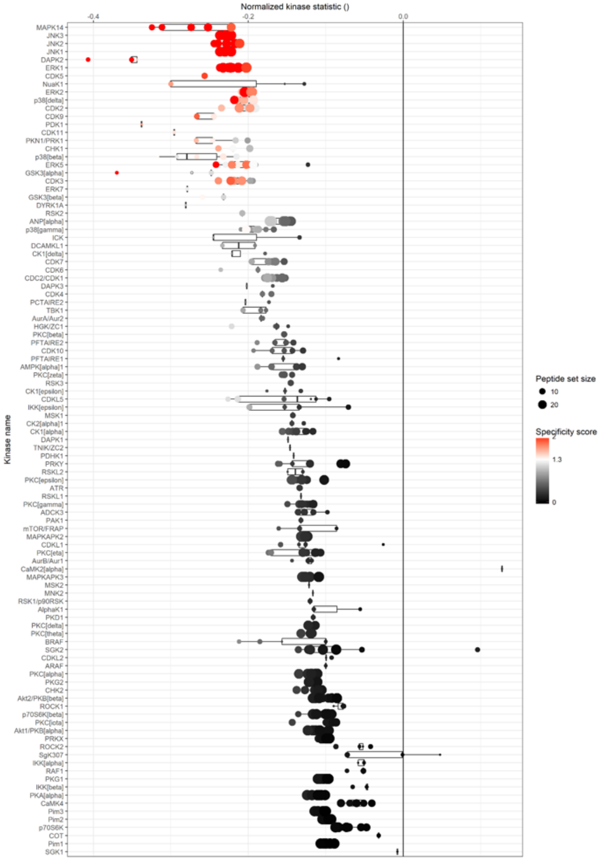


Rebastinib vs Veh

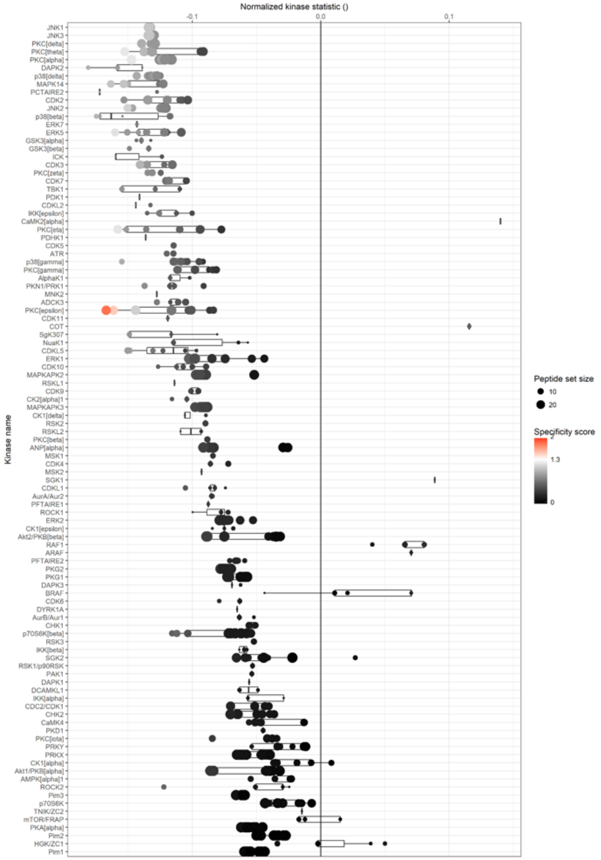


Olverembatinib vs Veh

HCC-F-4

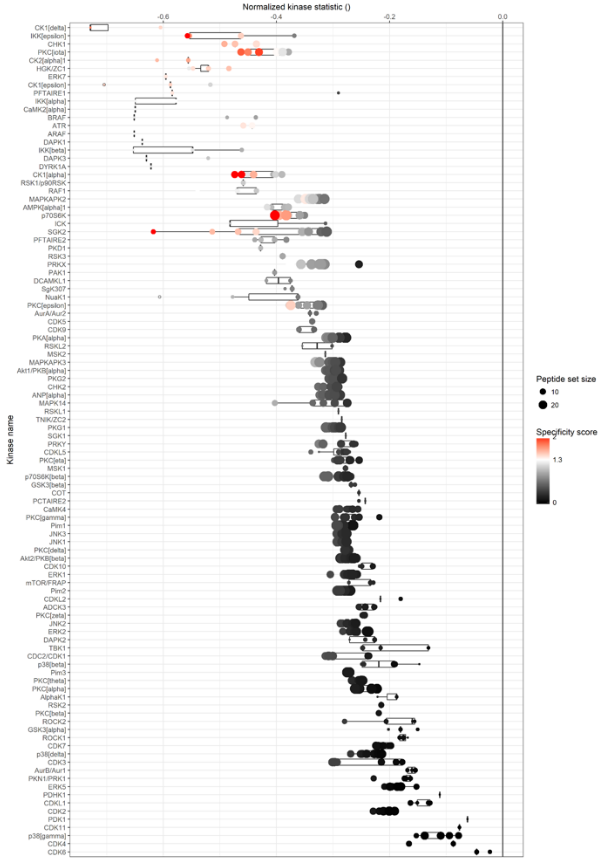


Imatinib vs Veh

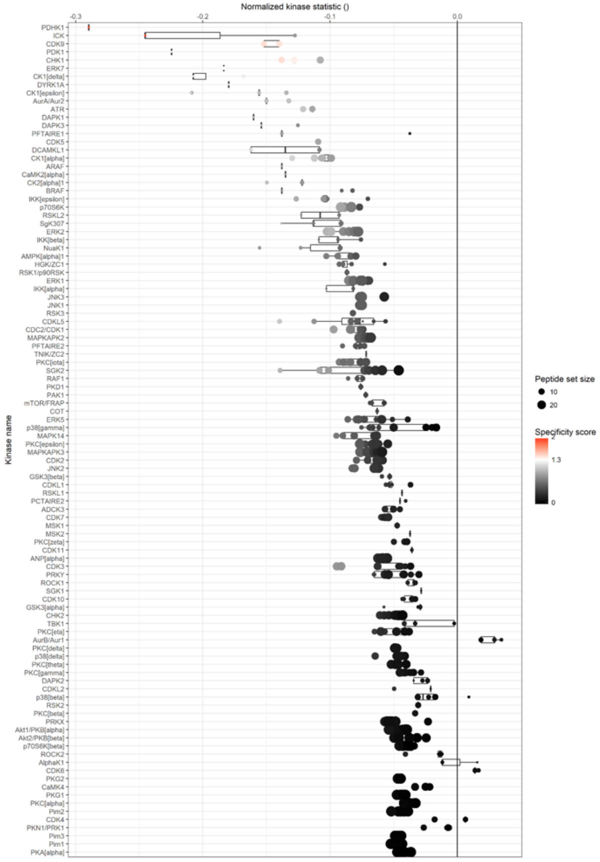


Rebastinib vs Veh

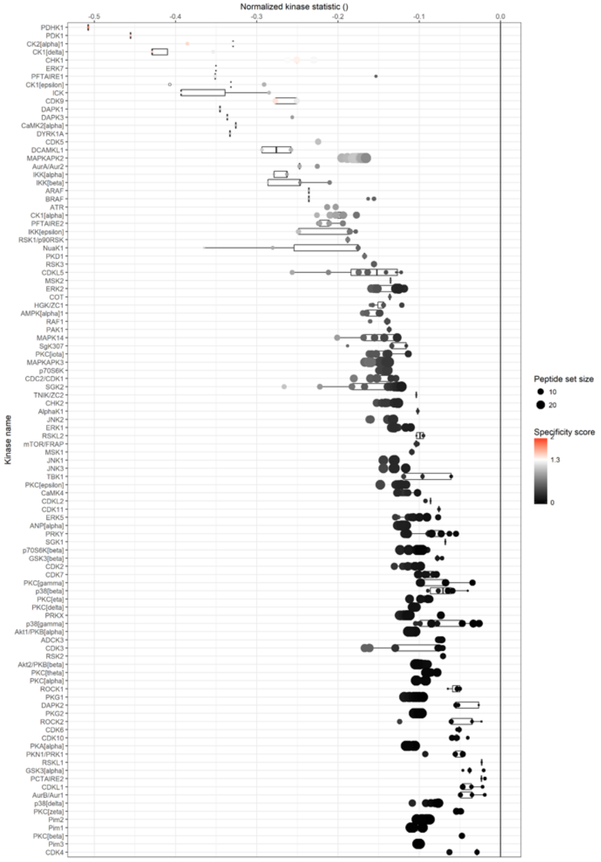


Olverembatinib vs Veh

HCC-M-5

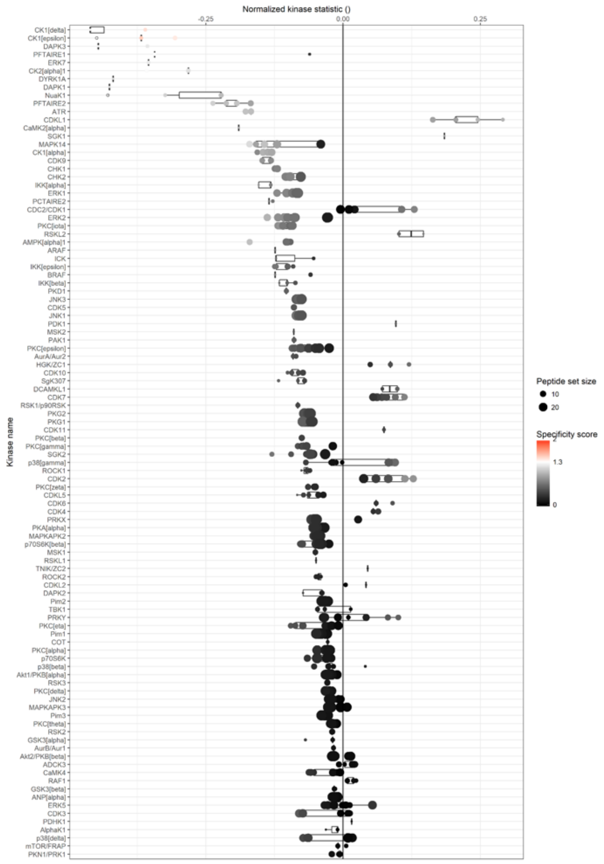


Imatinib vs Veh

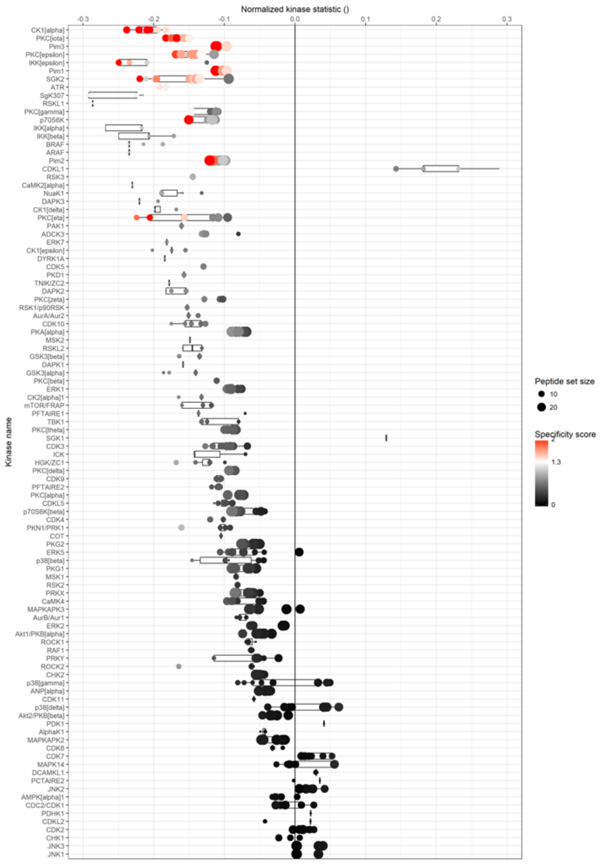


Rebastinib vs Veh

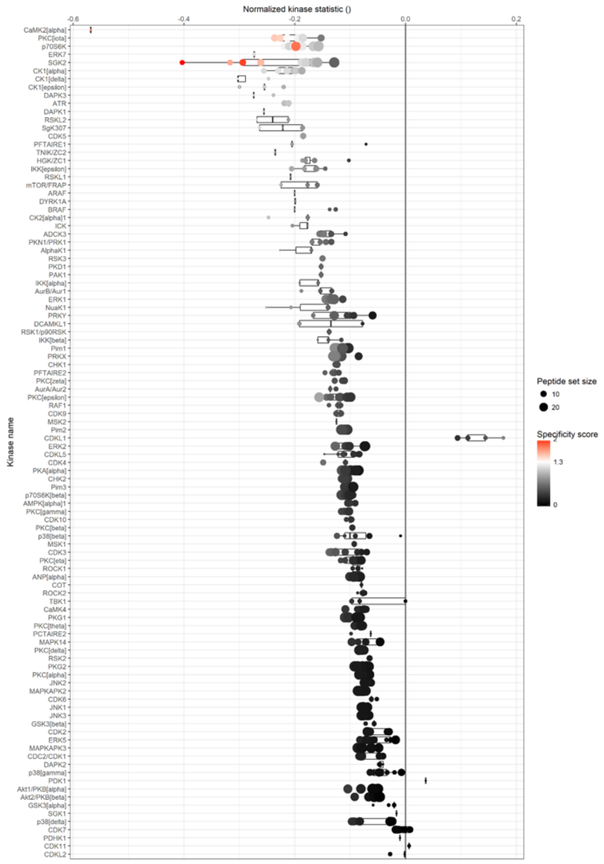


Olverembatinib vs Veh

HCC-F-6

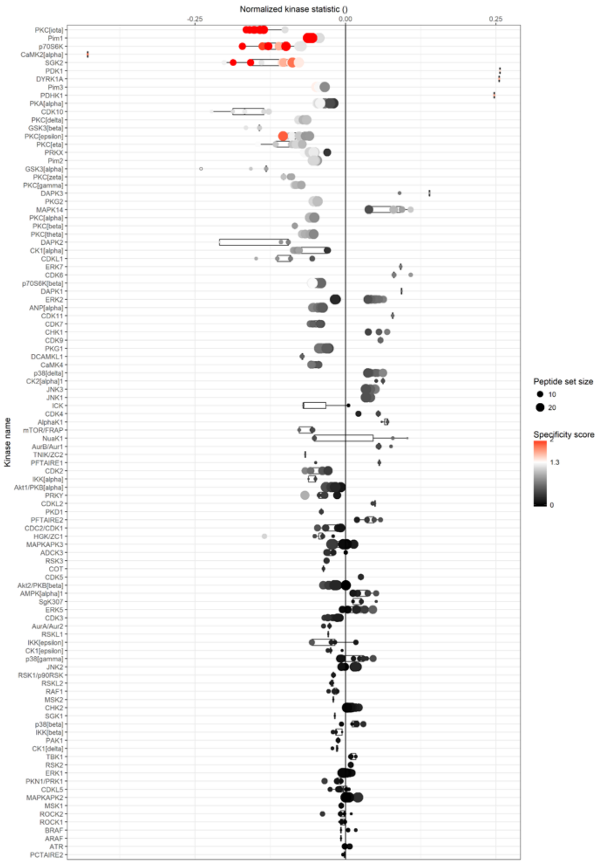


Imatinib vs Veh

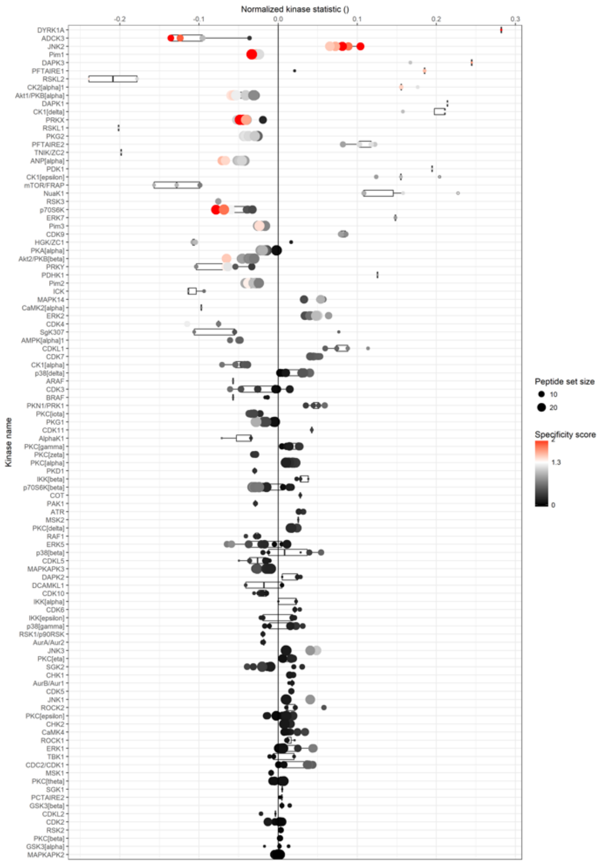


Rebastinib vs Veh

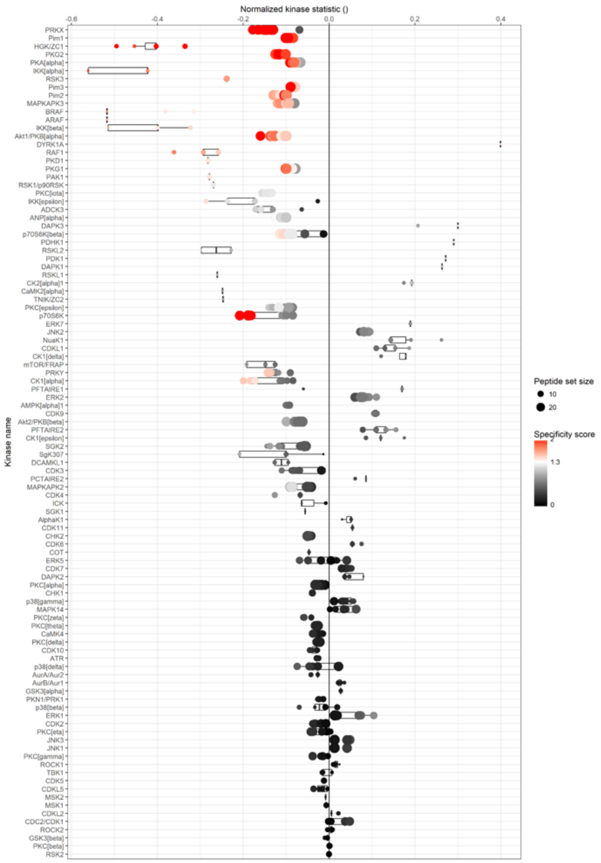


Olverembatinib vs Veh

HCC-F-7

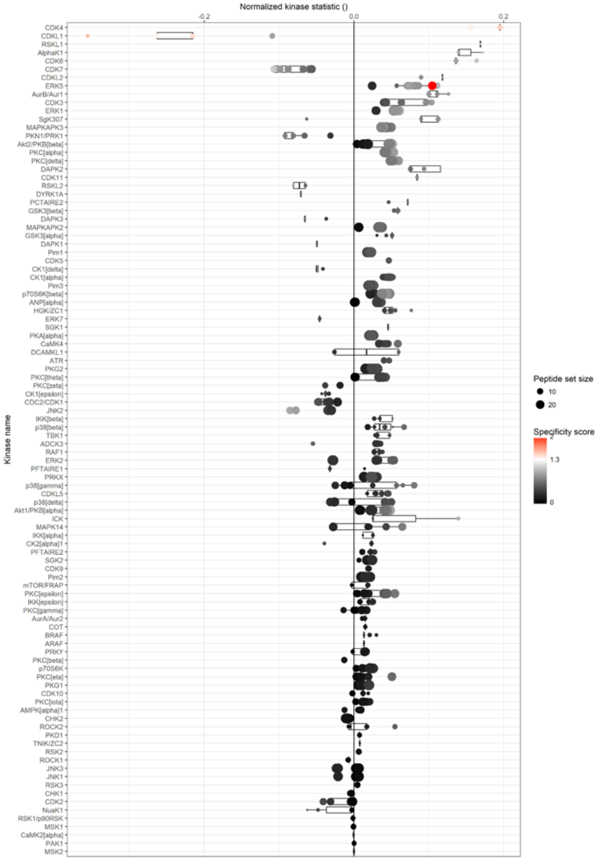


Imatinib vs Veh

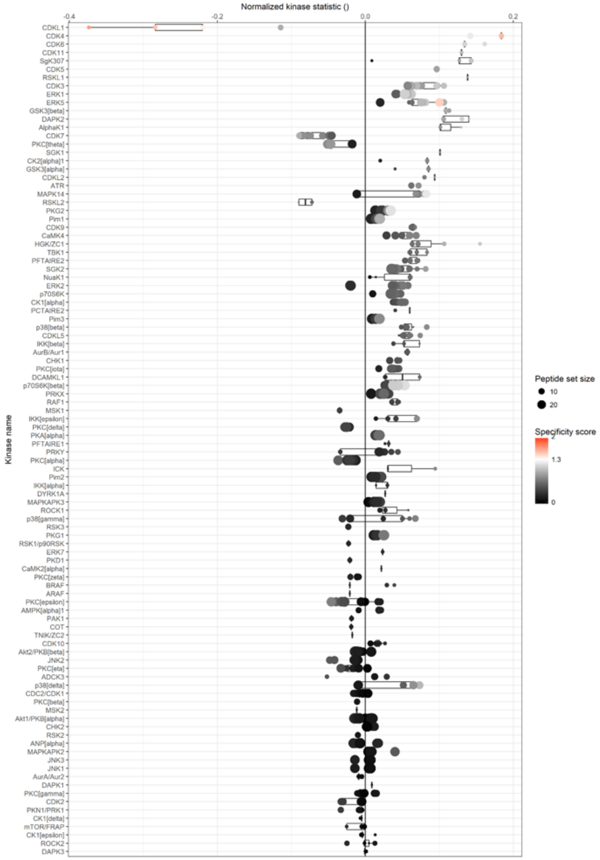


Rebastinib vs Veh

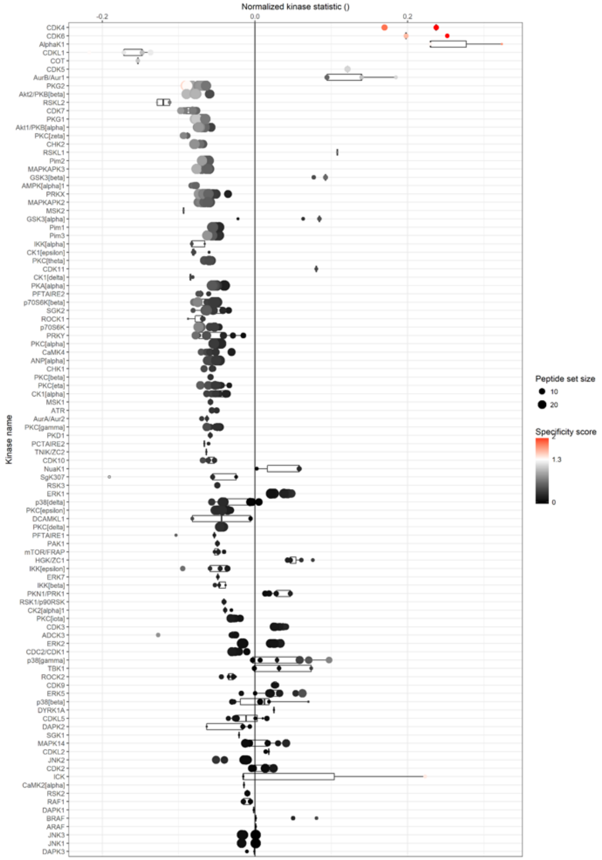


Olverembatinib vs Veh

HCC-M-8

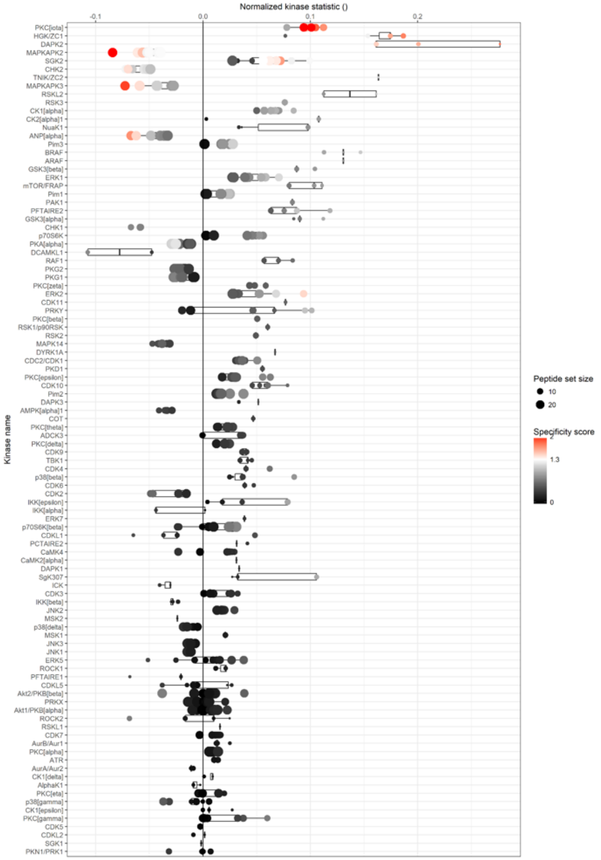


Imatinib vs Veh

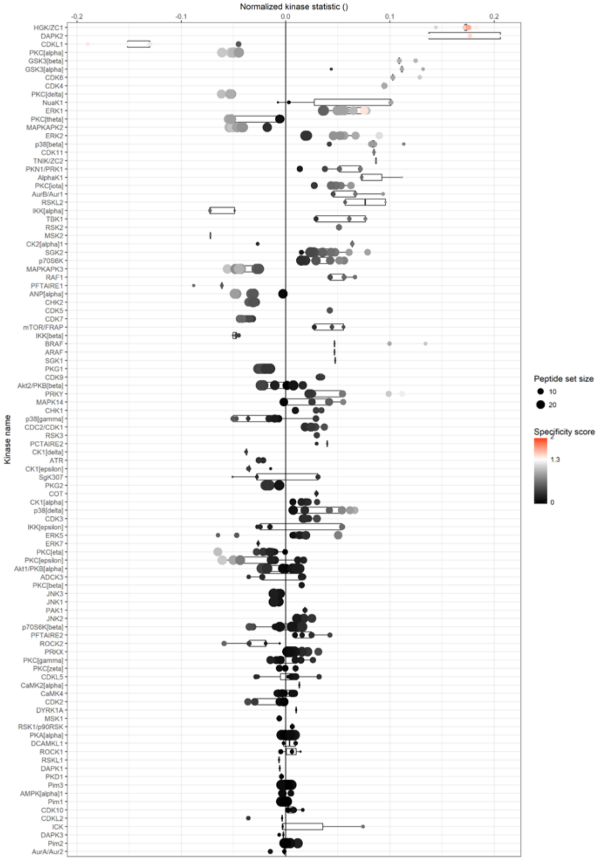


Rebastinib vs Veh

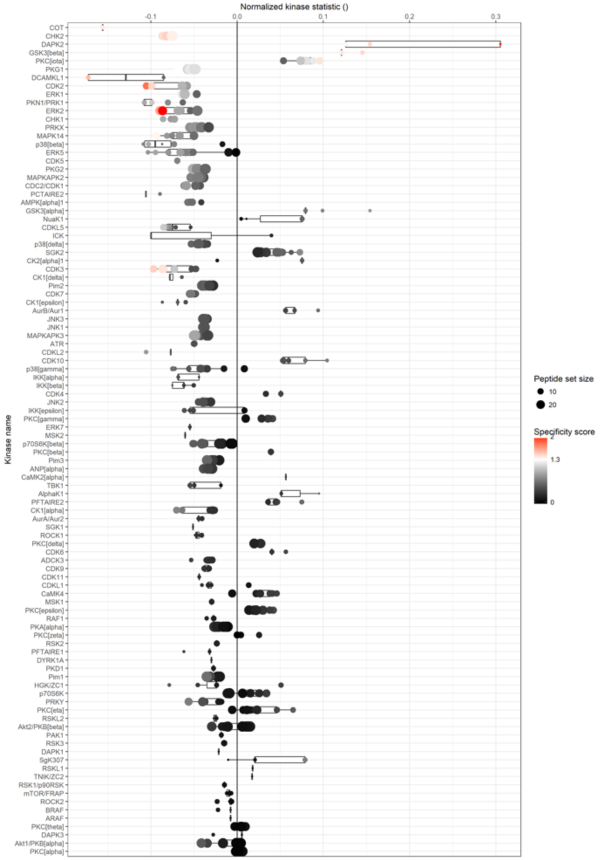


Olverembatinib vs Veh

# HCC-M-9

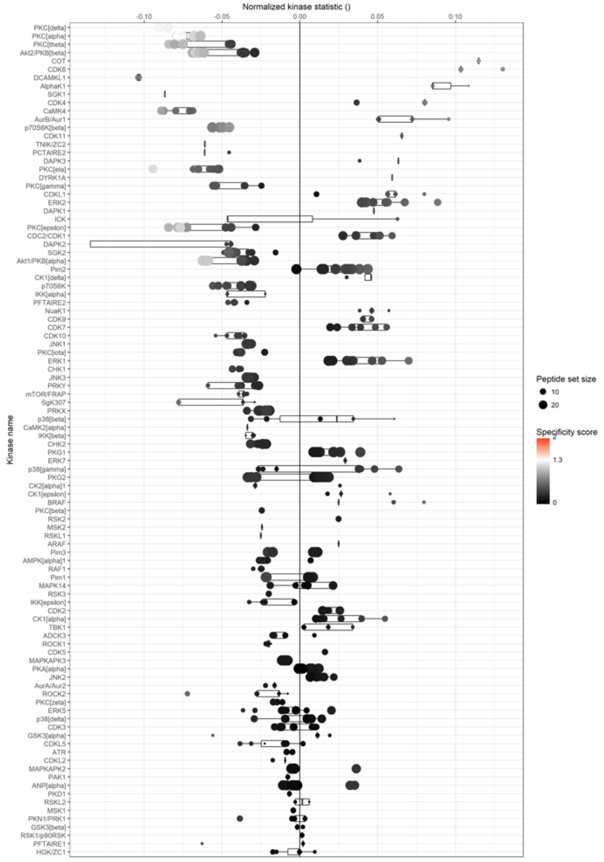


Imatinib vs Veh

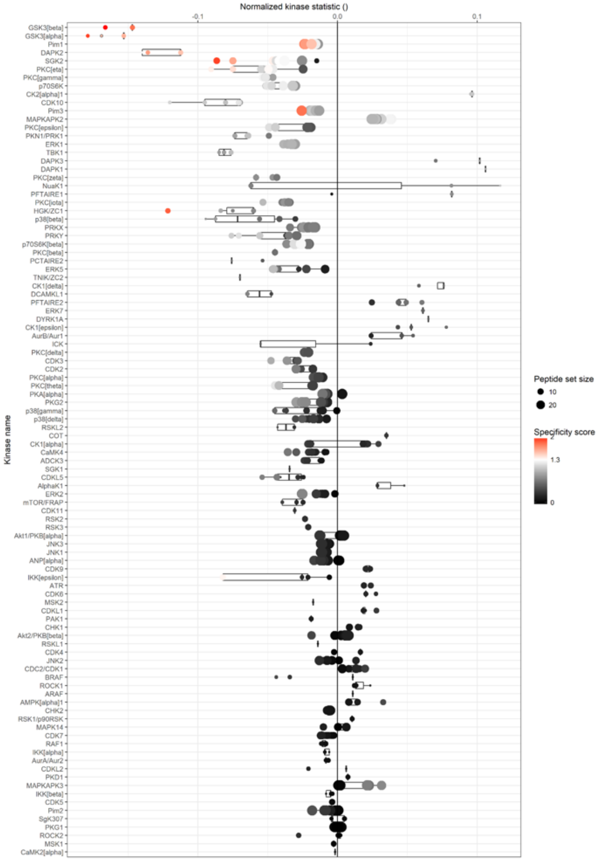


Rebastinib vs Veh

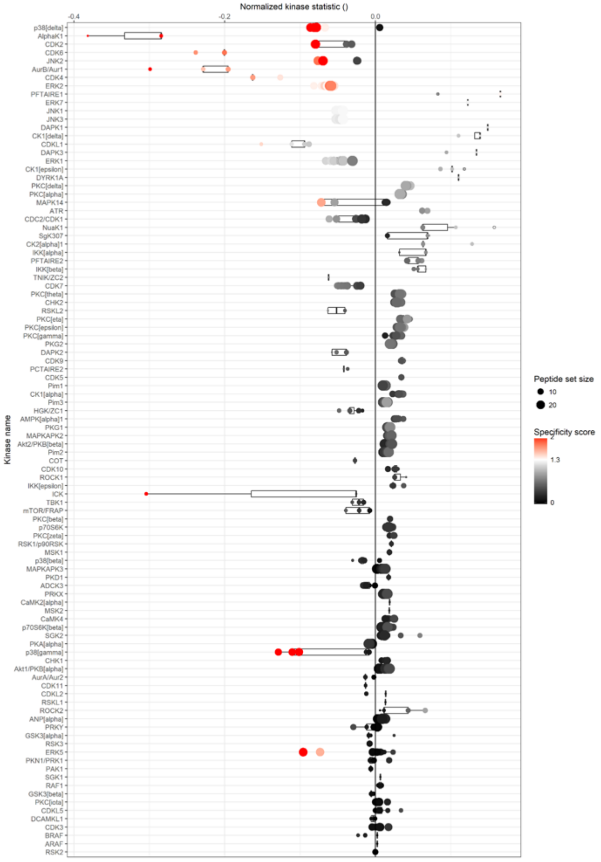


Olverembatinib vs Veh
