# Supplementary material for: Kinase signaling in liver disease via clinical-trial-on-a-PamChip: A distinctive methodology for drug mechanisms and personalized medicine
Source: J Biol Chem. 2026 Mar 18;302(5):111379. doi: 10.1016/j.jbc.2026.111379 (PMC13091354; doi:10.1016/j.jbc.2026.111379)
Supplement: Supplementary Material 4 [file mmc4.docx]

**STAR★METHODS**

**KEY RESOURCES TABLE**

| REAGENT or RESOURCE | SOURCE | IDENTIFIER |
| --- | --- | --- |
| Biological samples |  |  |
| Human Liver Biopsies of HCC Tumor and Normal Adjacent Tissue | University of Kentucky Markey Cancer Center Biospecimen Procurement & Translational Pathology Shared Resource Center | https://ukhealthcare.uky.edu/markey-cancer-center/research/srf/bptp |
| Chemicals, peptides, and recombinant proteins | | |
| DMEM/F-12 | Gibco | Cat#12500062 |
| DMEM | Gibco | Cat#10566016 |
| William’s Medium | Gibco | Cat#32551020 |
| Fetal Bovine Serum | Alkali Scientific | Cat#FB-72 |
| Anti-Anti | Gibco | Cat#15240062 |
| Hydrocortisone | Sigma-Aldrich | Cat#H0888 |
| Insulin Human | Sigma-Aldrich | Cat#I2643 |
| M-PER | Thermo Fisher Scientific | Cat#78501 |
| Protease Inhibitor Cocktail | Sigma-Aldrich | Cat#P2714 |
| Halt Phosphatase Inhibitor | Thermo Fisher Scientific | Cat#78420 |
| Dimethyl Sulfoxide (DMSO) | Sigma-Aldrich | Cat#472301 |
| Imatinib | Selleck Chemicals | Cat#S2475 |
| Rebastinib | Selleck Chemicals | Cat#S2634 |
| Olverembatinib | Selleck Chemicals | Cat#S7194 |
| QIAzol Lysis Reagent | Qiagen | Cat#79306 |
| Chloroform | Sigma-Aldrich | Cat#320331 |
| Ethanol 200 Proof | KOPTEC | DSP-MD-43 |
| TrueAMP SYBR Green qPCR SuperMix | Alkali Scientific | Cat#75013 |
| Critical commercial assays | | |
| Pierce Microplate BCA Protein Assay Kit | Thermo Fisher Scientific | Cat#23252 |
| Tyrosine Kinase PamChip-4 | PamGene | Cat#32516 |
| PTK Reagent Kit | PamGene | Cat#32112.5 |
| Serine/Threonine Kinase PamChip-4 | PamGene | Cat#32501 |
| STK Reagent Kit | PamGene | Cat#32201.3 |
| RNeasy Mini Kit | Qiagen | Cat#74106 |
| High Capacity cDNA Reverse Transcription Kit | Applied Biosystems | Cat#4368814 |
| Deposited data | | |
| Full Kinome Reports and Raw Kinome Files | This Paper | Figshare: DOI |
| Experimental models: Cell lines | | |
| HepG2 | Samir Softic | N/A |
| HLE | Robert Helsley | N/A |
| Huh-7 | Sadeesh Ramakrishnan | N/A |
| Hep3B2 | ATCC | LOT: 63194732 |
| HepaRG | Thermo Fisher Scientific | Cat#HPRGC10 |
| Oligonucleotides | | |
| Primers for RT-PCR, See Table S4 | This Paper | N/A |
| Antibodies | | |
| Rabbit polyclonal anti-c-Abl | Cell Signaling Technology | Cat#2862; RRID:AB_2257757 |
| Rabbit monoclonal anti- Phospho-c-Abl (Tyr245) | Cell Signaling Technology | Cat#2868; RRID:AB_2221094 |
| Software and algorithms | | |
| Evolve 3 | PamGene | https://pamgene.com |
| BioNavigator | PamGene | https://pamgene.com |
| RStudio | Posit Software | https://posit.co |
| KRSA | DePasquale et al. ^1^ | https://github.com/CogDisResLab/KRSA |
| CORAL | Metz et al. ^2^ | http://phanstiel-lab.med.unc.edu/CORAL/ |
| Prism 10.4 | GraphPad Software | https://www.graphpad.com |
| Other | | |
| PamStation 12 | PamGene | https://pamgene.com |

**EXPERIMENTAL MODEL AND STUDY PARTICIPANT DETAILS**

**Hepatocellular Carcinoma Samples**

For these studies, we used frozen human liver specimens from the University of Kentucky Markey Cancer Center Biorepository. All studies using human tissue samples conformed to the ethical guidelines of the 1975 Declaration of Helsinki, as reflected in approval by the Human Subjects Committee of the University of Kentucky College of Medicine. In brief, human liver biopsies of hepatocellular carcinoma and normal adjacent tissue were received from the Biospecimen Procurement & Translational Pathology Shared Resource Facility (BPTP SRF) of the University of Kentucky Markey Cancer Center (P30CA177558). The samples were deidentified from the research group and designated non-human research with IRB exemption. The H&E images in Figure 1 were provided by bptp srf. The clinical characteristics of the study population are in Table S1.

**Human cell lines**

HepG2 and Huh-7 cells were cultured in DMEM/F12 medium (Gibco, 12500062) supplemented with 10% fetal bovine serum (FBS, Alkali Scientific, FB-72) and 1% Anti-Anti (AA, Gibco, 15240062). HLE and Hep3B2 cells were cultured in DMEM (Gibco, 10566016) supplemented with 10% FBS and 1% AA. HepaRG cells were cultured in Williams medium (Gibco, 32551020) supplemented with 10% FBS, 1% AA, 24.7 μg/mL hydrocortisone (Sigma-Aldrich, H0888), and 5 μg/mL insulin (Sigma-Aldrich, I2643). All cells were grown at 37° °C with 5% CO_2_ in a humidified incubator.

**METHOD DETAILS**

**PamGene PamStation Kinome Analysis**

Kinase activity was quantified in the HCC and normal adjacent tissue samples using the PamGene PamStation 12 instrument, as previously described ^3-7^. Samples were cut and pooled within their respective group for figures 1-7 and processed individually for figures 8, S2, and S3. Samples were lysed using Qiagen TissueLyser and M-PER protein extraction reagent (Thermo Fisher Scientific, 78501) supplemented with protease inhibitor (Sigma-Aldrich, P2714) and Halt phosphatase inhibitor (Thermo Fisher Scientific, 78420). The samples were centrifuged, and the supernatant was transferred to a new tube. Protein concentration was determined by Pierce Microplate BCA Protein Assay Kit (Thermo Fisher Scientific, 23252). The samples were diluted to a 2.5 μg/μL concentration in lysis buffer. Per array for STK PamChip (PamGene, 32501), 1 μg of protein was used, and 5 μg was used for PTK PamChip (PamGene, 32516). The lysates were added to the PamChip in the presence of 4 mM adenosine triphosphate (ATP) and fluorescently labeled antibodies (PamGene, 32112.5 PTK, 32201.3 STK) to detect the phosphorylation of 196 (PTK) or 144 (STK) peptide substrates per array. The samples were run in technical triplicate using 3 PamChips per PTK and STK. Evolve 3 (PamGene) software uses a charge-coupled device (CCD) camera and light-emitting diode (LED) imaging system to record relative phosphorylation levels of each unique consensus phosphopeptide sequence every 5 min for 60 min as measured by peptide signal intensities recorded across 10, 20, 50, and 100 millisecond exposure times over 94 cycles for PTK and 124 cycles for STK. Raw imaging data were exported for further data analysis, and kinase mapping was described further in the BioNavigator Bioinformatics Analysis and Kinome Random Sampling Analysis (K.R.S.A) section.

**Clinical Trial in a PamChip**

Protein lysate from the men and women HCC tumor region prepared above from the kinome analysis was used to perform the clinical trial in a PamChip. A protein lysis method similar to that described above was used for the individual HCC samples. For the HepG2, Huh-7, HLE, Hep3B2, and HepaRG validation runs, protein was harvested 24 hours after a change to the normal culture medium described above. The cells were scraped, and protein was extracted using M-PER supplemented with protease and phosphatase inhibitors as described above, and cell pellets were homogenized using a pellet pestle. A protocol similar to that described above for the PamGene PamStation Kinome Analysis was used, with two modifications. The same amount of protein lysate described above was used. The protein lysates and run basic mix were incubated with either vehicle (DMSO, Sigma-Aldrich, 472301), Imatinib (1 μM) (Selleckchem, S2475), Rebastinib (100 nM) (Selleckchem, S2634), or Olverembatinib (100 nM) (Selleckchem, S7194) for 30 minutes at room temperature while rocking prior to the addition of the ATP and fluorescently labeled antibody. The concentrations were determined using the IC_50_ concentrations (provided in the table below) published on Selleckchem.com. The same final DMSO concentration (2%) was used across all treatments. The ATP concentration was reduced to 1 mM to prevent it from outcompeting the inhibitors used. The run conditions were the same as above. The raw images were exported for quantification and further analysis, as described below.

| Selleckchem.com Inhibitor IC_50_ Concentrations | |
| --- | --- |
| Inhibitor | IC_50_ |
| Imatinib | 0.6 μM |
| Rebastinib | 0.8 nM |
| Olverembatinib | 0.34 nM |

**BioNavigator Bioinformatics Analysis**

Images of the arrays obtained during the runs were analyzed using BioNavigator (PamGene). The across-chip coefficient of variation (CV) of the high signals for the technical replicates was within PamGene specifications, indicating low variability across chips. The CVs for the high-signal groups are shown in the table below for the pooled analysis. The fold change (FC) for each phosphopeptide was calculated using the signal ratios and averaged across the three PamChips. The minimum threshold cutoffs were determined from previous literature ^3-7^. These thresholds require differential phosphopeptide signals greater than or equal to 30% (FC ≥ 1.30 or FC ≤ 0.70) for differential phosphorylation to be considered. Linear regression slopes provide time-series phosphorylation intensity signals used to compare groups (e.g., experimental vs. control). Data was exported from BioNavigator after calculation of signal intensity for further data analysis. Upstream kinase identification was performed using BioNavigator Upstream Kinase Analysis (UKA) (PamGene). The UKA analysis identifies the kinases most likely responsible for phosphorylation events occurring on the PamChip over time. BioNavigator uses a mapping file containing experimentally confirmed and predicted substrates for each kinase, ranked by confidence from 0 (high confidence based on in vitro or in vivo experiments) to 12 (low confidence based on predictive models). The ABL substrates used for the heatmaps and their corresponding rankings are shown in **Supplemental Table S5**. The log-transformed signal intensity for the substrates was determined relative to normal adjacent tissue or vehicle and used for the PerMed plots and the ABL substrates heatmap. The kinase activity plots were made using Prism (GraphPad). For the kinome analysis of HCC tissue, data were analyzed by comparing the HCC tumor region with adjacent normal tissue for each sex. For the Clinical Trial in a PamChip, data were analyzed by comparing each inhibitor with the vehicle for each sex across liver samples, individual HCC samples, and cell lines.

| Coefficient of Variation of High Signals (Top 5%) | | |
| --- | --- | --- |
| Groups | PTK | STK |
| Male Normal Adjacent | 2% | 11% |
| Male HCC | 1% | 9% |
| Female Normal Adjacent | 2% | 14% |
| Female HCC | 2% | 6% |

**Kinome Random Sampling Analysis (K.R.S.A.)**

The signal intensity and saturation data were exported into an Excel file for further analysis using the Kinome Random Sampling Analysis (K.R.S.A) package on RStudio. Undetectable and/or nonlinear (R^2^ < 0.80) phosphopeptides are excluded from subsequent analyses. The data were analyzed by comparing the HCC tumor region with the adjacent normal tissue in liver samples. For the Clinical Trial in a PamChip, the data were analyzed by comparing each inhibitor to the vehicle for each sex for the liver samples, individual HCC samples, and cell lines. Full kinome reports and raw data files are available on Figshare (DOI TBD).

**CORAL Kinome Phyla Trees**

The kinome phyla tree was made using CORAL^2^. The phyla tree values were generated and exported from BioNavigator. Each node represents a kinase, and kinases are grouped by family. Only kinases measured in our analysis are included on the tree. The node size for each kinase refers to the mean final score for the direction of kinase activity. The node color represents the median kinase statistic, reflecting the magnitude of changes in kinase activity. Value ranges for node color and node size are based on the ranges of all values in the analysis and are consistent for comparison with the other phyla trees in the figure.

**RNA Extraction and Quantitative Real-Time PCR**

RNA was extracted from the HCC and the normal adjacent tissues for gene expression quantification using RT-PCR. The tissues were lysed in QIAzol (Qiagen, 79306) and homogenized using Qiagen TissueLyser. The RNA was extracted using phenol:chloroform separation by the addition of chloroform (Sigma-Aldrich, 320331) to the QIAzol. The upper supernatant was transferred to a new tube containing ethanol (KOPTEC, DSP-MD-43). Total RNA was purified using the RNeasy Mini Kit (Qiagen, 74106) according to the manufacturer’s instructions. The total RNA was quantified using a NanoDrop 2000 spectrophotometer (Thermo Fisher Scientific). Using total RNA, cDNA was synthesized using High Capacity cDNA Reverse Transcription Kit (Applied Biosystems, 4368814). RT-PCR was used to amplify and quantify the cDNA gene expression using specific primers (see Table S4) and TrueAMP SYBR Green qPCR SuperMix (Alkali Scientific, 75013). The thermocycling conditions consisted of: 95°C for 10 minutes, 60 cycles of 95°C for 15 seconds, 60°C for 30 seconds, and 72°C for 0-30 seconds based on primer product size and finished with a melt curve with temperatures ranging from 60-95°C to determine primer specificity. Normalization was performed in a separate reaction to RPL41. The RT-PCR graphs were made using Prism (GraphPad).

**Immunohistochemistry (IHC) Staining**

Immunohistochemistry was performed on the female and male HCC and the adjacent normal tissues by the Biospecimen Procurement & Translational Pathology Shared Resource Facility (BPTP SRF) of the University of Kentucky Markey Cancer Center (P30CA177558). Tissue sections were cut at 4 µm and mounted on positively charged slides. Slides were dried for at least 1 hour at 58 °C. Slides were stained on Ventana Discovery Ultra, with onboard deparaffinization and antigen retrieval, or by hand, with deparaffinization and hydration on an automated stain line and antigen retrieval in a Biocare Medical decloaking chamber, as indicated in the chart below. Conditions for each antibody were optimized using known or expected positive and negative control tissue, and staining was verified by a board-certified pathologist prior to staining the study set. All staining was accompanied by positive control tissue sections to confirm stain performance. Photo-scanning was performed with a Zeiss Axioscan Z1 slide scanner.

| Antibody | Catalog # | Dilution | Antigen Retrieval | Primary Incubation | Secondary | Visualization | Staining Platform |
| --- | --- | --- | --- | --- | --- | --- | --- |
| ABL | Cell Signaling  (2862) | 1:100 | Ventana CC1 standard (95°C, 64') | 37°C for 1 hour | Ventana OmniMap Rabbit HRP | Ventana ChromoMap DAB | Ventana Discovery Ultra |
| p-ABL (TYR^245^) | Cell Signaling  (2868) | 1:50 | Dako Low pH TRS,  (95°C, 20') | 4°C overnight | Dako Rabbit Link, 20'; Dako Flex-HRP, 20' | Dako DAB | Manual |

**QUANTIFICATION AND STATISTICAL ANALYSIS**

All RT-PCR graphs contain an individual point per sample (n # shown in Table S1) on the graph. The kinase activity graphs have a gray diamond for each kinase target substrate measured. The PamChip images are representative of one of the three chips run in the run. RT-PCR statistics were calculated using a Wilcoxon Test on Prism. P-values < 0.05 were considered statistically significant.

1. DePasquale, E.A.K., Alganem, K., Bentea, E., Nawreen, N., McGuire, J.L., Tomar, T., Naji, F., Hilhorst, R., Meller, J., and McCullumsmith, R.E. (2021). KRSA: An R package and R Shiny web application for an end-to-end upstream kinase analysis of kinome array data. PLoS One *16*, e0260440. 10.1371/journal.pone.0260440.

2. Metz, K.S., Deoudes, E.M., Berginski, M.E., Jimenez-Ruiz, I., Aksoy, B.A., Hammerbacher, J., Gomez, S.M., and Phanstiel, D.H. (2018). Coral: Clear and customizable visualization of human kinome data. bioRxiv, 330274. 10.1101/330274.

3. Badmus, O.O., Kipp, Z.A., Bates, E.A., da Silva, A.A., Taylor, L.C., Martinez, G.J., Lee, W.H., Creeden, J.F., Hinds, T.D., Jr., and Stec, D.E. (2023). Loss of hepatic PPARalpha in mice causes hypertension and cardiovascular disease. Am J Physiol Regul Integr Comp Physiol *325*, R81-R95. 10.1152/ajpregu.00057.2023.

4. Bates, E.A., Kipp, Z.A., Lee, W.H., Martinez, G.J., Weaver, L., Becker, K.N., Pauss, S.N., Creeden, J.F., Anspach, G.B., Helsley, R.N., et al. (2024). FOXS1 is Increased in Liver Fibrosis and Regulates TGFbeta Responsiveness and Proliferation Pathways in Human Hepatic Stellate Cells. J Biol Chem, 105691. 10.1016/j.jbc.2024.105691.

5. Bates, E.A., Kipp, Z.A., Martinez, G.J., Badmus, O.O., Soundarapandian, M.M., Foster, D., Xu, M., Creeden, J.F., Greer, J.R., Morris, A.J., et al. (2023). Suppressing Hepatic UGT1A1 Increases Plasma Bilirubin, Lowers Plasma Urobilin, Reorganizes Kinase Signaling Pathways and Lipid Species and Improves Fatty Liver Disease. Biomolecules *13*. 10.3390/biom13020252.

6. Creeden, J.F., Kipp, Z.A., Xu, M., Flight, R.M., Moseley, H.N.B., Martinez, G.J., Lee, W.H., Alganem, K., Imami, A.S., McMullen, M.R., et al. (2022). Hepatic kinome atlas: An in-depth identification of kinase pathways in liver fibrosis of humans and rodents. Hepatology *76*, 1376-1388. 10.1002/hep.32467.

7. Zelows, M.M., Cady, C., Dharanipragada, N., Mead, A.E., Kipp, Z.A., Bates, E.A., Varadharajan, V., Banerjee, R., Park, S.H., Shelman, N.R., et al. (2023). Loss of carnitine palmitoyltransferase 1a reduces docosahexaenoic acid-containing phospholipids and drives sexually dimorphic liver disease in mice. Mol Metab *78*, 101815. 10.1016/j.molmet.2023.101815.
